# Epitranscriptomic RNA m^6^A Modification in Cancer Therapy Resistance: Challenges and Unrealized Opportunities

**DOI:** 10.1002/advs.202403936

**Published:** 2024-12-11

**Authors:** Mohammad Burhan Uddin, Zhishan Wang, Chengfeng Yang

**Affiliations:** ^1^ Department of Pharmaceutical Sciences North South University Bashundhara Dhaka 1229 Bangladesh; ^2^ Stony Brook Cancer Center Stony Brook University Stony Brook NY 11794 USA; ^3^ Department of Pathology Renaissance School of Medicine Stony Brook University Stony Brook NY 11794 USA

**Keywords:** cancer stem cell, cancer therapy resistance, epitranscriptomics, m^6^A modification, m^6^A regulator inhibitors

## Abstract

Significant advances in the development of new cancer therapies have given rise to multiple novel therapeutic options in chemotherapy, radiotherapy, immunotherapy, and targeted therapies. Although the development of resistance is often reported along with temporary disease remission, there is often tumor recurrence of an even more aggressive nature. Resistance to currently available anticancer drugs results in poor overall and disease‐free survival rates for cancer patients. There are multiple mechanisms through which tumor cells develop resistance to therapeutic agents. To date, efforts to overcome resistance have only achieved limited success. Epitranscriptomics, especially related to m^6^A RNA modification dysregulation in cancer, is an emerging mechanism for cancer therapy resistance. Here, recent studies regarding the contributions of m^6^A modification and its regulatory proteins to the development of resistance to different cancer therapies are comprehensively reviewed. The promise and potential limitations of targeting these entities to overcome resistance to various anticancer therapies are also discussed.

## Graphical abstract

Tumors develop resistance to therapeutic agents, unfortunately, efforts to overcome resistance have achieved limited success. m^6^A RNA modification dysregulation is an emerging mechanism for cancer therapy resistance. This review comprehensively discusses recent studies regarding the contributions of m^6^A modification and its regulators to cancer therapy resistance and underlying mechanisms. The promise and limitations of targeting these entities to overcome resistance are also discussed.

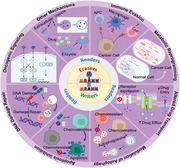

## 1 Background

The term, epitranscriptome, first coined a decade ago by Saletore et al. refers to the presence of base modifications in RNA molecules located at different transcript regions.^[^ ^1^ ^]^ Sitting between the genetic material of the cell, the DNA, and the translation of genetic traits into proteins, RNA molecules play important roles in the regulation of biological processes. Similar to epigenetic regulation of gene expression by DNA base modifications or epiproteomic changes in protein molecules, epitranscriptomic modification of RNA plays critical roles in the regulation of gene expression which is vital to normal cellular activities^[^ ^2^ ^]^ as well as pathogenesis in diseases.^[^ ^3^ ^]^ Chemical modifications occurring in the four common bases as well as rare bases in all forms of RNAs account for more than 170 modification types to date.^[^ ^4^ ^]^ Among these, N^6^‐methyladenosine (m^6^A) is the most abundant internal modification in both coding and non‐coding RNAs. Although discovered in the early 1970s,^[^ ^5^ ^]^ the pathophysiologic role of m^6^A has only recently been elucidated, particularly its role in cancer.^[^ ^6^ ^]^ Over the past ten years, extensive literature reported on m^6^A modification in cancer.^[^ ^7^ ^]^

Cancer is one of the leading causes of morbidity and mortality globally and is therefore a leading health crisis for several decades.^[^ ^8^ ^]^ Carcinogenesis, due to the uncontrolled proliferation of otherwise normal cells of the body, is a complex multifaceted crisis inside the cell or tissue. Failure to surgically remove all deranged cells is insufficient for cure and has spurred scientists to dive deep into the cancer development process. This has resulted in the development of treatment modalities ranging from chemotherapy to irradiation to targeted therapies. The history of treatment with chemotherapeutic agents or radiation therapy for the treatment of cancer is quite lengthy. Conventional cancer chemotherapeutic drugs (e.g., antimetabolites, alkylating agents, cytotoxic antibiotics, steroid hormone analogs or microtubule inhibitors) have been used for more than half a century.^[^ ^9^ ^]^ Unfortunately, resistance to these conventional therapies is a major impediment to effective cancer treatment. The response to resistance to monotherapy was initially polychemotherapy (also known as combination therapy); this is still a popular practice.^[^ ^10^ ^]^ The emergence of multidrug resistance in cancer cells poses new challenges in combating cancer and is ushering in the development of therapy to target those molecules or pathways that divert a normal cell toward malignancy, i.e., targeted therapy; as a result, entirely new types of anticancer drugs have emerged including tyrosine kinase inhibitors,^[^ ^11^ ^]^ intra‐ or extracellular receptor inhibitors or the inhibitors of molecules involved in cellular signal transduction pathways.^[^ ^10^, ^12^ ^]^ Recent innovations have shown promise — the use of immunologic approaches (e.g., monoclonal antibodies^[^ ^13^ ^]^) targets specific molecules or exploits the patients’ own immune system for recognition and eradication of cancer cells, for example, modifying patient's own T cells^[^ ^14^ ^]^ using genetic engineering.^[^ ^7^, ^15^ ^]^ Despite these successes, there are reports of the emergence of resistance to anticancer therapies indicating that there is more to unravel to improve our understanding of molecular mechanisms associated with cancer therapy resistance. The observation that m^6^A RNA modification is an underlying cause of cancer therapy resistance is revolutionizing cancer therapy development. In this review, we report on recent discoveries involving m^6^A RNA modification in cancer therapy resistance and provide updates on the development of new molecules targeting this modification and its regulators in overcoming resistance.

## 2 Regulation of m^6^A RNA Modification: “Writing”, “Reading”, or “Erasing” the Crypt

**Figure 1:**
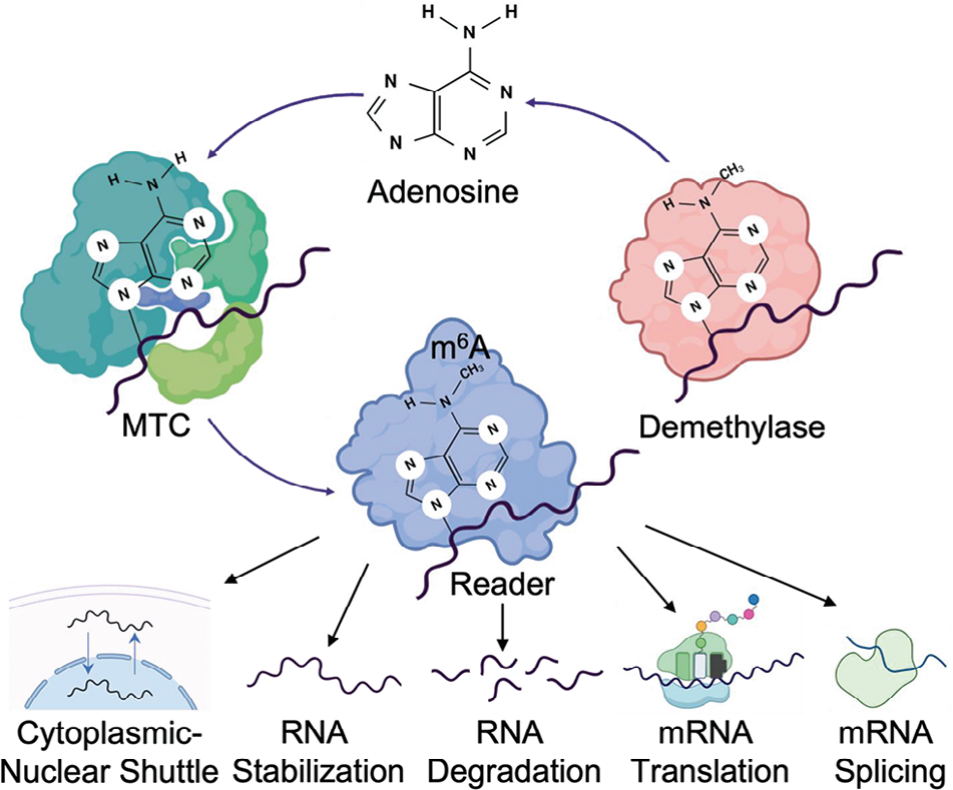
m^6^A modification regulators (Writers, Erasers, and Readers) and their roles in RNA fate determination. MTC: Methyltransferase complex; Writers (METTL3, METTL14, WTAP, RBM15, RBM15B, VIRMA/KIAA1429, HAKAI, ZC3H13, METTL16); Erasers (FTO, ALKBH5); Readers (YTHDF1‐3, YTHDC1‐2, hnRNPC, hnRNPA2B1, eIF3, IGF2BP1‐3, ELAVL1/HuR, FMRP).

m^6^A modification is not a permanent modification transcript signature; rather it is dynamic in nature, engraved through a group of proteins named “writers” of a methyltransferase complex (MTC) and erased by another type of enzyme called “erasers”. The effects of the modification, however, are not evident unless recognized by a third group of proteins named “readers”. As a widespread modification in mRNAs and other non‐protein‐coding RNAs like long non‐coding RNAs (lncRNAs), microRNAs (miRNAs), circular RNAs (circRNAs), etc., m^6^A regulates the fate and function of the RNAs (**Figure** **1**).^[^ ^16^ ^]^ Researchers have observed m^6^A modification in the consensus sequence motif RRACH (where the first R  represents A, G, or U; the second R represents A or G and H represents A, U, or C) in mRNAs; this occurs most abundantly in places like internal exons, near the stop codon or the 3′‐UTR regions.^[^ ^17^ ^]^ The specific factor(s) that ensure these site‐specific localizations of m^6^A in the transcriptome were reported in recent studies revealing the presence of exon junction complex (EJC), a group of m^6^A deterrent proteins that prevent this modification at exon junctions and their proximities.^[^ ^18^ ^]^ In these transcripts, EJC creates a physical barrier for the MTC preventing them from binding to their target sequence motifs that fall within a range of ≈100 nucleotides from the splice junctions and carrying out the modification.^[^ ^18a^ ^]^ This is particularly significant for short internal exons where the presence of EJC hinders the installation of m^6^A even in the presence of sequence motif, while average‐length to long internal exons can overcome this barrier; thus, emphasizing the effect of exon architecture on site‐specificity and overall m^6^A topology.^[^ ^18c^ ^]^ This site‐specific localization of m^6^A signifies its role in mRNA stability or translation, playing vital functions in various biological processes.^[^ ^18^ ^]^ Methylation of adenosine (A) nucleotide at N‐6 position is brought about by the complex of methyltransferases (writers) which contains methyltransferase like‐3 (METTL3), methyltransferase like‐14 (METTL14), Wilms’ tumor 1‐associating protein (WTAP), RNA‐binding motif protein15 (RBM15), RBM15B, Vir‐like m^6^A methyltransferase associated protein (VIRMA)/KIAA1429, E3 ubiquitin‐protein ligase Hakai (HAKAI) and zinc finger CCCH domain‐containing protein13 (ZC3H13). In this complex, METTL3 is the main catalytic protein containing the catalytic domain whereas METT14 has partial catalytic function. Other methyltransferases in this complex are necessary for the functional activity of the main catalytic proteins.^[^ ^16^, ^19^ ^]^ An independent m^6^A writer, METTL16, also catalyzes methyl transfer without being a member of the MTC complex.^[^ ^20^ ^]^ The reversible m^6^A modification in RNA transcripts is erased by demethylases Fat mass and obesity‐associated protein (FTO) and α‐ketoglutarate‐dependent dioxygenase alkB homolog5 (ALKBH5). Both FTO and ALKBH5 belong to the non‐heme Fe^2+^ and α‐ketoglutarate‐dependent dioxygenase family that catalyze m^6^A removal in a two‐step reaction requiring oxygen and involving oxidation of Fe^2+^ to Fe^3+^, generating adenosine.^[^ ^21^ ^]^ A group of m^6^A‐binding proteins recognize the presence of m^6^A on the RNA molecule, exhibit the effect of the modification on the transcript, and determine the biological role of the m^6^A modification in the organism. A number of m^6^A‐recognizing proteins have been identified as m^6^A readers, including YTH domain‐containing family proteins1‐3 (YTHDF1‐3), YTH domain‐containing proteins1‐2 (YTHDC1‐2), heterogeneous nuclear ribonucleoprotein C and A2B1 (hnRNPC, hnRNPA2B1), eukaryotic initiation factor3 (eIF3), insulin‐like growth factor‐2 mRNA binding proteins1‐3 (IGF2BP1‐3), ELAVL1/Hu antigen R (HuR) and fragile X mental retardation protein (FMRP).^[^ ^16^, ^22^ ^]^ Subcellular localization and the dynamic interactions of these m^6^A‐regulatory proteins determine the ultimate fate of the modified transcript. While the writers and erasers are mainly located in the cellular nuclei, the distribution of readers is across the cytoplasmic and nuclear regions. Depending on recognition by a specific reader, the stability/degradation, shuttles between the cytoplasmic and nuclear region, translation and splicing (mRNA), or maturation (miRNA) of the RNA molecule is determined.^[^ ^23^ ^]^ Although it has been speculated that, each of the reader proteins plays a definitive role in determining mRNA fate, recent studies have shown functional redundancies among some readers, e.g., YTHDF1‐3. YTHDF1, and YTHDF3 stabilize the transcripts and promote their translation,^[^ ^24^ ^]^ while YTHDF2 induces mRNA decay.^[^ ^25^ ^]^ Contrary to these findings, several others observed redundant functions of these YTH‐proteins in which they bind with the same m^6^A‐modified transcript to cause their decay instead of enhancing translation.^[^ ^26^ ^]^ The possible reason for such discrepancies may be because of the differential expressions of these reader proteins in different tissue types. There could possibly be an implication of site‐specific localization of m^6^A modification due to the presence of EJC that changes m^6^A topology in the same target transcripts in different individual cell types.^[^ ^18^ ^]^ This overall can affect the fate of the transcripts differentially in different tissues, for instance, a healthy versus a cancerous tissue. These functional discrepancies among m^6^A regulatory proteins indicate their diverse roles in a context‐dependent fashion.

## 3 Phenotypic Attributes Associated with m^6^A RNA Modification‐Mediated Resistance Development

**Figure 2:**
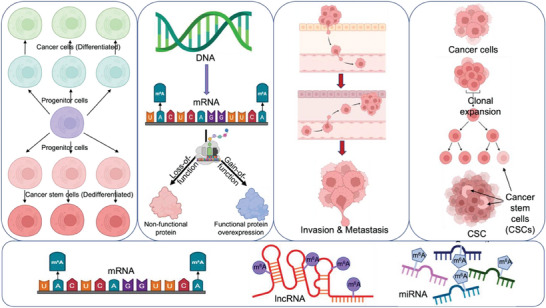
Phenotypic changes in cancer cells associated with m^6^A RNA modification. Abnormal m^6^A levels in the RNA transcripts due to aberrant expression of m^6^A regulators are responsible for cancer cell dedifferentiation, loss/gain‐of‐function, invasiveness, and cancer stem cell (CSC) generation.

Extensive literature has documented the association of m^6^A modification and its regulators with phenotypic attributes in cancer cells that contribute to the development of resistance to conventional therapeutic agents used (**Figure** **2** and **Table** **1**).

**Table 1:** m^6^A regulators associated with phenotypic attributes of cancer cells contribute to therapeutic resistance.

Cancer Type	m^6^A Regulator(s)	Phenotypic Attribute(s)	Clinical Outcomes	Target
Glioblastoma	↑ METTL3, HuR	Dedifferentiation Increased stem cell‐like properties	Reduced radiosensitivity Poor patient survival	SOX2^[^ ^28a^ ^]^
	↓ METTL3 ↓METTL14 ↑FTO	GSC differentiation GSC self‐renewal	Glioblastoma progression Alters lifespan in experimental animals	ADAM19^[^ ^31^ ^]^
	↑ ALKBH5	Enhanced tumorigenicity of GSC	Poor patient prognosis	FOXM1^[^ ^47^ ^]^
	↑ YTHDF2	GSC generation and maintenance	Glioblastoma development and progression	MYC, VEGFA^[^ ^51^ ^]^
Acute myeloid leukemia (AML)	↑ METTL3	Reduced myeloid differentiation AML cell proliferation	Leukemia progression	c‐MYC, BCL2, PTEN^[^ ^28b^ ^]^
	↑ METTL14	Reduced myeloid differentiation Leukemia stem cell self‐renewal	AML development	MYB, MYC^[^ ^29^ ^]^
	↑METTL16	Increased leukemia stem/initiating cells (LSCs/LICs)	Leukemogenesis	BCAT1‐2^[^ ^43^ ^]^
	↑ FTO	Dedifferentiation Increased leukemia stem/initiating cells (LSCs/LICs)	Oncogenic cell transformation Leukemogenesis	ASB2, RARA^[^ ^32^ ^]^; MYC^[^ ^33^, ^34^ ^]^
	↑ ALKBH5	LSCs/LICs self‐renewal	Poor AML prognosis	TACC3^[^ ^48^ ^]^
	↑ YTHDF2	LSC development and integrity	Leukemic transformation, leukemogenesis and propagation	Tnfrsf2 (TNFR2)^[^ ^52^ ^]^
	↑ IGF2BP2	Leukemia stem/initiating cells (LSCs/LICs) self‐renewal	Enhances AML development Poor AML prognosis	MYC, GPT2, SLC1A5^[^ ^44^ ^]^ PRMT6^[^ ^45^ ^]^
Colorectal cancer (CRC)	↑ METTL3	Enhanced metastasis; increased CRC self‐renewal and stemness	Poor cancer prognosis	SOX2 [36a]circ1662^[^ ^36c^ ^]^
	↑ METTL3 ↑ IGF2BP1	Increased CRC stemness and chemoresistance	Poor cancer prognosis	Sec62^[^ ^41^ ^]^
	↑ METTL3 hnRNPA2B1	Increased metastasis and chemoresistance	CRC progression	TCF7L2^[^ ^37^ ^]^
Lung adenocarcinoma (LUAD)	↑ METTL3	Increased lung cancer proliferation and invasion	Poor cancer prognosis	circKRT17^[^ ^105^ ^]^
	↑ METTL3 YTHDF2	Increased stem‐like cells leading to chemoresistance	Poor overall and disease/progression‐free survival	TUSC7^[^ ^115^ ^]^
Hepatocellular carcinoma (HCC)	↑METTL3	Loss of p53 function	Shorter overall patient survival	p53^[^ ^76^ ^]^
	↑METTL3 ↑IGF2BP1	Promotes HCC proliferation, invasiveness, and metastasis	Poor overall and disease‐free patient survival	NIFK‐AS1^[^ ^39^ ^]^
	↓ METTL14 IGF2BPs	Decreased HCC differentiation and CSC generation; malignant transformation	Poor HCC prognosis and patient survival	HNF3γ^[^ ^30^ ^]^
	↑IGF2BP1	Increased HCC proliferation, invasiveness, and metastasis	Poor HCC patient survival	circMDK^[^ ^38^ ^]^
Pancreatic cancer (PC)	↓ALKBH5	Loss of p53 function	Poor clinicopathological manifestations	PER1^[^ ^40^ ^]^
Breast cancer	↓ALKBH5	BCSC enrichment	Increases mortality	NANOG^[^ ^49^ ^]^
Oral squamous cell carcinoma (OSCC)	↑ALKBH5	Enhanced stemness associated gene expression	Decreases overall patient survival	FOXM1 NANOG^[^ ^50^ ^]^
Ovarian cancer	↑YTHDF1	Increased cancer stem cell‐like phenotype	Poor patient prognosis	TRIM29 [53]

### 3.1 Dedifferentiation/Plasticity of Cancer Cells

A cancerous cell has to withstand hostile conditions for its survival such as oxygen and nutrient deprivation or immune assault; thus, it must dedifferentiate from its surrounding tissues or adopt cellular plasticity. Altered translational efficiency through mRNA modification plays vital roles in cellular dedifferentiation or conferring plasticity to cancer cells.^[^ ^27^ ^]^ As the presence or absence of m^6^A modification in the mRNA transcripts regulates their stability and translation, aberrant expression of m^6^A regulators plays a critical role in determining cancer cell differentiation and plasticity. METTL3 overexpression has been implicated in reduced differentiation of tumorigenic glioma cells and acute myeloid leukemia (AML) cells.^[^ ^28^ ^]^ Enhanced METTL3 expression inhibits differentiation of hematopoietic stem/progenitor cells (HSPCs) whereas METTL3 depletion enhances differentiation and stalled progression of AML cells. At the molecular level, mRNA transcripts of cellular Myc (c‐MYC), B cell lymphoma‐2 (BCL2), and phosphatase and tensin homolog (PTEN) are assumed to be the target of METTL3. Increased m^6^A methylation enhances translation of these genes promoting AML progression.^[^ ^28b^ ^]^ Another important member of the m^6^A writer complex, METTL14 is downregulated during normal myeloid differentiation, which is overexpressed in AML cells blocking myeloid differentiation and promoting AML development. MYB and MYC transcripts have been identified as the downstream target of METTL14, increasing their stability and translation.^[^ ^29^ ^]^ METTL14 is associated with hepatocellular carcinoma (HCC) cell dedifferentiation and cancer stem cell (CSC) generation conferring resistance to chemotherapeutics. Hepatocyte nuclear factor 3γ (HNF3γ) is the target of METTL14, which is upregulated during hepatic differentiation, but decreased in dedifferentiated cancerous cells. METTL14 incorporates m^6^A into HNF3γ mRNA which is recognized by IGF2BP family readers (IGF2BP1, IGF2BP2, and IGF2BP3); this results in stabilization and upregulation of HNF3γ mRNA during hepatocyte differentiation. In dedifferentiated HCC cells, METTL14 is downregulated, with decreased levels of HNF3γ mRNA halting cancer cell differentiation.^[^ ^30^ ^]^ METTL3 overexpression is associated with enhanced cellular differentiation in glioblastoma. METTL3 and METTL14 knockdown or FTO overexpression favors tumor progression in glioblastoma due to altered m^6^A levels in the target gene, ADAM metalloproteinase domain 19 (ADAM19).^[^ ^31^ ^]^ A contrasting finding, also reported by Li et al., showed that FTO‐mediated demethylation of the target oncogenic mRNA transcripts results in poor differentiation of AML cells leading to oncogenic cell transformation and leukemogenesis. FTO overexpression in AML cells causes demethylation and downregulation of ASB2 and RARA which are responsible for AML cell differentiation.^[^ ^32^ ^]^ A similar proleukemogenic role for FTO is also reported in another study in which it is associated with the dedifferentiation of leukemic cells through demethylation and stabilization of MYC mRNA.^[^ ^33^ ^]^ Pharmacologic inhibition or knockdown of FTO also promotes myeloid differentiation of AML cells.^[^ ^34^ ^]^ SLC12A5, a neuron‐specific potassium chloride cotransporter (K+/Cl‐ cotransporter) plays a non‐neuronal protumorigenic role in chemoresistance in castration‐resistant prostate cancer through neuroendocrine differentiation of cancerous cells. SLC12A5, by complexation with YTHDC1, upregulates HOXB13 which is required for neuroendocrine differentiation and chemoresistance. YTHDC1, in this case, binds with the m^6^A on HOXB13 and stabilizes the transcript thereby causing its upregulation.^[^ ^35^ ^]^

### 3.2 Invasiveness and Metastasis

Higher METTL3 expression was observed in metastatic cancerous tissues in colorectal cancer (CRC) patients and is responsible for poor disease prognosis.^[^ ^36^ ^]^ SOX2 is identified as the downstream target of METTL3, which increases m^6^A levels in the coding regions of SOX2 mRNA. The m^6^A reader IGF2BP2 recognizes the modification and increases its levels by inhibiting degradation and thus facilitating the metastatic spread of cancer.^[^ ^36a^ ^]^ Circular RNA has been proposed as another target for METTL3 in the metastatic spread of CRC. METTL3 installs m^6^A to circ1662 inducing its expression which further activates the YAP1/SMAD3 pathway causing EMT of CRC cells and thus enhanced invasiveness and metastasis.^[^ ^36c^ ^]^ METTL3‐mediated m^6^A modification and hnRNPA2B1 recognition enhances TCF7L2 expression which further activates Wnt/β‐catenin signaling and facilitates the invasiveness and metastatic spread of CRC.^[^ ^37^ ^]^ METTL3 also introduces m^6^A in microRNA miR‐1246 which activates the MAPK pathway and is also responsible for the invasiveness and metastasis of CRC cells.^[^ ^36b^ ^]^ Another m^6^A reader IGF2BP1 is upregulated in HCC cells which binds with the m^6^A site on the circular RNA circMDK, stabilizing and upregulating its expression. circMDK upregulation further activates the PI3K/Akt/mTOR pathway and promotes the proliferation, invasiveness, and migration of HCC cells.^[^ ^38^ ^]^ In another study, METTL3‐mediated m^6^A modification on lncRNA NIFK‐AS1 is recognized by IGF2BP1 causing its stabilization which further activates Akt1. Akt1 activation upregulates the membrane metalloproteinases MMP‐7 and MMP‐9 promoting HCC metastasis.^[^ ^39^ ^]^

### 3.3 Cancer Stem‐Like Cell Self‐renewal/Pluripotency

Stem cell‐like properties of cancer cells are a key feature needed to confer chemoresistance. METTL3 is associated with enhanced stemness of CRC cells via upregulation of stemness‐associated genes and proteins. Sec62 is a known modulator of stem cell markers that regulates the stem‐like behavior of CRC cells. In CRC cells, METTL3 enhances the m^6^A abundance on Sce62 mRNA which is read by IGF2BP1 thereby increasing its stability for regulation of stemness behavior of the cancer cells and multidrug resistance.^[^ ^41^ ^]^ METTL3 overexpression has been implicated in reduced differentiation of tumorigenic glioma cells (also associated with the stem cell attributes of glioblastoma stem‐like cells (GSCs)). The SOX2 mRNA appears to be the target of METTL3 which inserts the m^6^A modification to the 3′‐UTR on the SOX2 mRNA. The RNA reader HuR is also necessary for the stabilization of SOX2 mRNA which is responsible for its overexpression resulting in stem cell‐like properties of the glioblastoma cells.^[^ ^28a^ ^]^ METTL3 is also associated with enhanced stem‐like cells in CRC via the introduction of m^6^A in the SOX2 mRNA, enhancing its stability.^[^ ^36a^ ^]^ In clear cell renal cell carcinoma (ccRCC) METTL3 upregulation increases the cancer stem‐like cell population.^[^ ^42^ ^]^

Metabolic reprogramming is an important target for conveying self‐renewal capability to cancer stem or initiating cells. METTL16, an independent methyltransferase, which deposits m^6^A methylation to the target transcripts without being a part of the methyltransferase complex, is an essential component for the self‐renewal of LSCs/LICs (leukemia stem/initiating cells). METTL16 incorporates m^6^A modification into branched‐chain amino acid transaminases (BCAT1 and BCAT2), upregulating their expression thereby causing metabolic reprogramming of AML cells as well as maintaining LSC/LIC self‐renewal.^[^ ^43^ ^]^ The m^6^A‐binding protein, IGF2BP2, an m^6^A reader, promotes the self‐renewal capability of LSCs/LICs by modulating the metabolic programming of the cells. In one study, investigators observed that IGF2BP2 increases LSCs by enhancing the stability and translation of genes such as MYC, GPT2, and SLC1A5 and altering glutamine uptake and metabolism in AML cells^[^ ^44^ ^]^; in another study, IGF2BP2 stabilized protein arginine methyltransferase (PRMT6) mRNA thereby interfering with lipid transport and metabolism and facilitating LSC/LIC maintenance.^[^ ^45^ ^]^ c‐Myc induction and increased stemness and chemoresistance have also been observed in gastric cancer due to enhanced c‐Myc mRNA stability by m^6^A modification.^[^ ^46^ ^]^ Aberrant expression of m^6^A demethylases FTO and ALKBH5 has been observed in AML which contributes to LSC/LIC maintenance and self‐renewal. FTO is overexpressed in LSCs/LICs implicating it in self‐renewal and stem cell‐like properties in LSCs. FTO knockdown or pharmacological inhibition efficiently reduced LSC populations in murine AML models.^[^ ^34^ ^]^ ALKBH5 overexpression is associated with increased GSC and LSC/LIC populations. ALKBH5 demethylates the 3′‐UTR of the nascent transcripts of the transcription factor FOXM1 protecting it from m^6^A recognition and degradation by HuR, thus stabilizing and enhancing FOXM1 expression in patient‐derived GSCs.^[^ ^47^ ^]^ ALKBH5, on the other hand, targets and regulates expression of TACC3 which plays an important role in LSC/LIC self‐renewal.^[^ ^48^ ^]^ A contrasting effect of ALKBH5 has been observed in breast cancer stem cell (BCSC) enrichment due to the hypoxic stress of breast cancer cells. Exposure to hypoxic conditions enhances expression of ALKBH5 that demethylates the pluripotency factor NANOG mRNA at the 3′‐UTR region, thereby upregulating its expression and contributing to the BCSC development.^[^ ^49^ ^]^ ALKBH5‐mediated overexpression of NANOG and FOXM1 leads to an increased stem cell population and cisplatin resistance in oral squamous cell carcinoma (OSCC). ALKBH5 demethylates the 3′‐UTR of FOXM1 and NANOG pre‐mRNA, increasing their expression and thus enhancing the CSC population which contributes to chemoresistance and cancer recurrence.^[^ ^50^ ^]^ Increased m^6^A modification was also identified in GSCs compared to normal neuronal stem cells (NSCs). It is not clear which m^6^A writer is associated with this upregulation; however, the m^6^A reader, YTHDF2, has been reported to be responsible for the stemness of GSCs via stabilizing pluripotency‐associated transcripts MYC and VEGFA.^[^ ^51^ ^]^ YTHDF2 is also upregulated in AML and is responsible for maintaining the self‐renewal properties and overall integrity of LSCs via degradation and downregulation of the Tnfrsf2 transcript which expresses the tumor necrosis factor receptor 2 (TNFR2).^[^ ^52^ ^]^ Increased m^6^A and YTHDF1 have been identified in ovarian cancer cells that are responsible for stem cell‐like properties and resistance to cisplatin. Although the involvement of a specific writer is not clear in the setting of increased levels of m^6^A, its modification at the 3′‐UTR site of the TRIM29 is read by YTHDF1, increasing its translation and overexpression. Increased TRIM29 is responsible for the stemness and cisplatin resistance to the cancer cells.^[^ ^53^ ^]^ The m^6^A reader hnRNPA2B1 is overexpressed in gastric cancer (GC) cells which enhances stemness properties through stabilization of lncRNA NEAT1 and activation of Wnt/β‐catenin pathway, thus conferring resistance to multiple chemotherapeutic agents including Adriamycin (doxorubicin), Vincristine and 5‐FU.^[^ ^54^ ^]^

## 4 Molecular Mechanisms of Multidrug Resistance by m^6^A RNA Modification and Its Regulators

**Figure 3:**
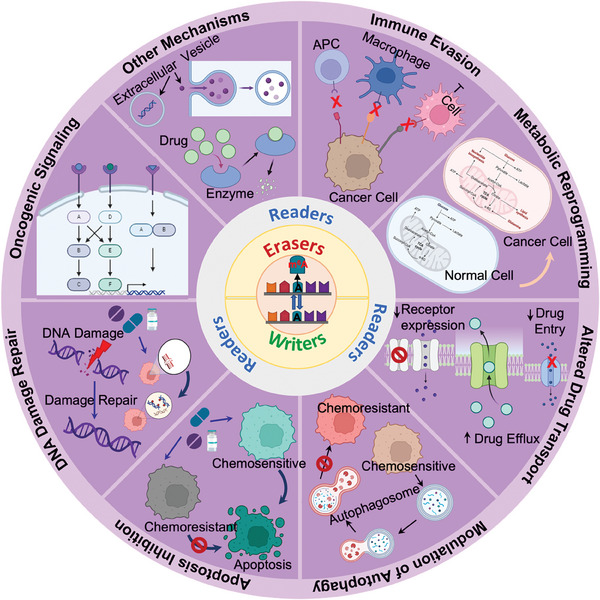
Molecular mechanism of m^6^A modification‐mediated multidrug resistance in cancer cells. Abnormal expressions of m^6^A regulators alter the m^6^A landscape in the target transcripts, expressing molecules that are associated with drug transport inside the cells, metabolic processes, programmed cell death, autophagy, DNA damage repair, protumorigenic cellular signaling or immune infiltration in the TME which confer resistance to various therapeutic agents. TME: Tumor microenvironment.

The implications of m^6^A methylation and its regulators in resistance to different classes of commonly used cancer therapeutics are summarized in **Table** **2** Mechanisms are discussed in this section (**Figure** **3**).

**Table 2:** m^6^A‐mediated resistance mechanisms against different therapeutic classes.

Resistance Against	Cancer Type	Associate m^6^A Regulator(s)	Underlying Cause	Molecular Target (s)	Mechanism	Reference
**Antimetabolites**						
Gemcitabine	Pancreatic ductal adenocarcinoma (PDAC)	↓ALKBH5	Activation of Wnt signaling	WIF1	ALKBH5 downregulation and increased m^6^A on WIF1 mRNA decreases its expression and diminishes its inhibition on Wnt signaling pathway	[113]
Gemcitabine	Pancreatic cancer	↑METTL14	Enzymatic inactivation of gemcitabine	CDA	METTL14 enhances gemcitabine and inactivates cytidine deaminase (CDA)	[101]
Arabinocytosine (AraC)	Acute myeloid leukemia (AML)	↑METTL3	Inhibition of apoptosis	MYC	METTL3‐mediated MYC overexpression causes upregulation of proapoptotic and downregulation of antiapoptotic genes	[73]
**Cytotoxic antibiotics**						
Doxorubicin	Breast cancer	↑METTL3	Enhanced drug efflux	miR‐221‐3p	METTL3‐mediated m^6^A modification promotes miR‐221‐3p maturation that causes overexpression of MDR1 and ABCG2 (BCRP)	[58]
Doxorubicin	Breast cancer	↑FTO	Activation of STAT3 signaling	STAT3	FTO‐mediated decreased m^6^A modification of STAT3 transcript increases p‐STAT3 expression and signaling activation	[58]
Doxorubicin/Adriamycin	Breast cancer	IGF2BP2	Enhanced drug efflux	ABCB1	lncRNA A1BG‐AS‐mediated IGF2BP2 recruitment and m^6^A recognition on ABCB1 mRNA; ABCB1 mRNA stabilization	[61]
Doxorubicin/Adriamycin	Breast cancer	↑YTHDC1	DNA damage repair	BRCA1 RAD51	YTHDC1 induces DNA damage repair through BRCA1/RAD51 overexpression	[84]
Doxorubicin	TNBC	↑ALKBH5	Reduced cellular ROS	FOXO1	FOXO1 overexpression due to ALKBH5‐mediated demethylation and reduction of ROS	[85]
Doxorubicin	Colorectal cancer (CRC)	↑IGF2BP3	Enhanced drug efflux	ABCB1	Increases m^6^A and IGF2BP3‐mediated ABCB1 mRNA stabilization; ABCB1 overexpression	[63]
Doxorubicin	Acute myeloid leukemia (AML)	↑METTL3	Decreased autophagy	PTEN	m^6^A modification accelerates PTEN degradation resulting in protein expression that inhibits autophagy	[58]
**Alkylating agents**						
Carboplatin	Ovarian cancer	↑METTL3	Activation of cAMP‐dependent signaling	PTGER2	m^6^A‐induced PTGER2 upregulation results in increased self‐renewal and DNA damage repair	[111]
Cisplatin	Non‐small cell lung cancer (NSCLC)	↑METTL3	Activation of signaling through Akt	Akt1	Increased m^6^A modification in Akt1 mRNA and Akt1 upregulation	[108]
Cisplatin	Ovarian cancer	↑METTL3 IGF2BP1	Upregulation of PIK3R1 and PI3K/Akt activation	circPLPP4	m^6^A‐modified circPLPP4 upregulation increases PIK3R1 expression and PI3K/Akt activation	[109]
Cisplatin	Bladder cancer	↓YTHDC1	Activation of PI3K/Akt signaling	PTEN	YTHDC1 downregulation destabilizes PTEN causing PI3K/Akt activation	[110]
Cisplatin	Seminoma	↑METTL3 IGF2BP1	Activation of DNA damage repair genes	TFAP2C	m^6^A‐mediated TFAP2C stabilization activates DNA repair genes WEE1 and BRCA1	[82]
Cisplatin	Oral squamous cell carcinoma (OSCC)	↑ALKBH5	Increased CSC population by pluripotency regulatory gene expression	NANOG FOXM1	ALKBH5 removes m^6^A from FOXM1 and NANOG mRNA leading to CSC generation	[50]
Cisplatin	Bladder cancer	↑WTAP	Decreased cisplatin‐induced cell apoptosis	TNFAIP3	Increased m^6^A on TNFAIP3 increases its stability and expression which suppresses cell apoptosis	[75]
Cisplatin	NK/T cell lymphoma (NKTCL)	↑WTAP	Decreased apoptosis and increased drug efflux	DUSP6	m^6^A methylation of DUSP6 mRNA increases its expression which downregulates proapoptotic proteins and upregulates antiapoptotic proteins and drug efflux pumps	[60]
Cisplatin	Gastric cancer	METTL3‐METTL14‐WTAP	Decreased cisplatin‐induced cell apoptosis	c‐Myc	m^6^A modification enhances c‐Myc stability which increases stemness and chemoresistance	[46]
Cisplatin	Bladder cancer	↓METTL16	Reduced autophagy	PMEPA1	METTL16 downregulation destabilizes autophagy regulator PMEPA1	[71]
Cisplatin	Gastric cancer	↑FTO	Modulation of autophagy	ULK1	Decreased m^6^A methylation stabilizes and upregulates ULK1 which modulates autophagy	[72]
Cisplatin	Gastric cancer	METTL3 YTHDC1	Decreased ferroptosis	FAM120A	m^6^A modified FAM120A mRNA stabilization and upregulation inhibits cisplatin‐induced ferroptosis	[74]
Cisplatin	Colorectal cancer (CRC)	↑YTHDF1	Altered glutamine metabolism	GLS1	YTHDF1 promotes GLS1 expression resulting in increased glutamine uptake and metabolism	[88]
Cisplatin	Epithelial ovarian cancer (EOC)	↑ALKBH5	Activation of JAK2/STAT3 signaling	JAK2	ALKBH5 removes m^6^A from JAK2 protecting it from degradation which in turn activates STAT3	[112]
Cisplatin	Epithelial ovarian cancer	METTL3 YTHDF1	Activation of NK‐κB	RIPK4	METTL3‐mediated m^6^A installation and YTHDF1 recognition on RIPK4 mRNA prevents m^6^A degradation and NK‐κB activation resulting in cisplatin resistance	[116]
Cisplatin	Ovarian cancer	↑YTHDF1	Increased stem cell and resistant	TRIM29	m^6^A enrichment in TRIM29 increases its translation and is responsible for resistance	[53]
Oxaliplatin	Gastric cancer	↑METTL3	DNA damage repair	PARP1	Increased m^6^A enhances PARP1 stabilization leading to base excision repair	[81]
Oxaliplatin	Colorectal cancer (CRC)	↑METTL3	M2‐macrophage infiltration	TRAF5	m^6^A modification of TRAF5 mRNA decreases its abundance inhibiting oxaliplatin‐induced necroptosis	[98]
Oxaliplatin	Colorectal cancer (CRC)	↑YTHDF3	Overexpression of copper efflux transporter ATP7A and Hedgehog (HH)/GLI signaling activator DYRK1B	ATP7A DYRK1B	m^6^A modification causes ATP7A and DYRK1B upregulation resulting in oxaliplatin resistance	[65]
Temozolomide	Glioblastoma	↑METTL3	Repair DNA damage caused by temozolomide	MGMT APNG	m^6^A enrichment and upregulation of MGMT and APNG leads to repair of cytotoxic DNA damage	[83]
Temozolomide	Glioblastoma	↑FTO	Increased glucose metabolism	PDK1	PDK1 demethylation increases its stability thereby facilitating aerobic glycolysis	[86]
**Steroid hormones and their antagonists**						
Tamoxifen	Breast cancer	↓YTHDF2	Enhanced drug efflux	ABCB1	Decreased m^6^A upregulates ATF3; ATF3 enhances ABCB1 expression	[62]
Enzalutamide	Castration‐resistant prostate cancer	↑YTHDC1	Neuroendocrine differentiation of cancer cells	HOXB13	YTHDC1 complexing with SLC12A5 enhances HOXB13 transcript stability and differentiation	[35]
Dexamethasone	T cell acute lymphoblastic leukemia (T‐ALL)	↑ALKBH5	Inhibition of apoptosis	USP1	Reduced ALKBH5 mediated m^6^A increases USP1 which decreases dexamethasone‐induced apoptosis	[80]
**Protein kinase inhibitors**						
Imatinib	Gastrointestinal stromal tumor (GIST)	↑METTL3 YTHDF1 eEF‐1	Enhanced drug efflux	MRP1	Increased m^6^A‐mediated MRP1 mRNA translation	[57]
Erlotinib	Lung adenocarcinoma (LUAD)	↑METTL3 YTHDF2	Activation of Notch signaling	TUSC7	m^6^A modified TUSC7 degradation by YTHDF2 reverses TUSC7‐mediated Notch inhibition	[115]
Gefitinib	Non‐small cell lung cancer (NSCLC)	↑METTL3	Activation of autophagy	LC3 ATG5 ATG7	METTL3 positively regulates autophagy by m^6^A‐mediated upregulation of autophagy genes	[69]
Gefitinib	Non‐small cell lung cancer (NSCLC)	↑FTO YTHDF2	Enhanced drug efflux	ABCC10	Decrease in mRNA decay due to FTO‐mediated demethylation	[64]
Gefitinib	Lung adenocarcinoma (LUAD)	↑METTL3	Activation of Hippo signaling	lncRNASNHG17 (LATS2)	lncRNASNHG17 upregulation suppresses LATS2 resulting in gefitinib resistance	[106]
Osimertinib	Lung adenocarcinoma (LUAD)	↑METTL3	Activation of Hippo signaling	circKRT17 (YAP1)	m^6^A‐mediated circKRT17 stabilization increases YAP1 nuclear localization which promotes P‐gp and MRP‐1 expression	[105]
Apatinib	Hepatocellular carcinoma (HCC)	↑METTL3	Modulation of apoptotic gene expression	p53	METTL3‐catalyzed m^6^A modification and inactivation of p53 results in decreased apoptosis	[76]
Sunitinib	Renal cell carcinoma (RCC)	↑METTL14 IGF2BP2	Suppression of angiogenesis and apoptosis	TRAF1	m^6^A modification of TRAF1 transcript stabilization and TRAF1 upregulation suppresses proapoptotic protein expression	[78]
Sorafenib	Hepatocellular carcinoma (HCC)	↓METTL3	Activation of autophagy	FOXO3	Decreased m^6^A downregulates FOXO3 which in turn activates autophagy resulting in sorafenib resistance	[70]
Sorafenib	Hepatocellular carcinoma (HCC)	↑METTL3	Activation of MAPK/ERK pathway	DUXAP8	m^6^A modification upregulates lncRNA DUXAP8 which recruits miRNA miR‐584‐5p to activate MAPK1 resulting in chemoresistance	[114]
Sorafenib	Hepatocellular carcinoma (HCC)	↓METTl14 IGF2BPs	Reduced drug uptake	HNF3γ	Decreased m^6^A on HNF3γ mRNA causes its downregulation resulting in lower transcriptional expression of drug transporters	[30]
Sorafenib	Hepatocellular carcinoma (HCC)	↑METTL3 ↑IGF2BP1	Reduced drug uptake	NIFK‐AS1	m^6^A methylation‐regulated NIFK‐AS1 stabilization causes OATP1B1 and 3 downregulation	[39]
Sorafenib	Hepatocellular carcinoma (HCC)	MTC	Wnt/β‐catenin pathway activation	circRNA‐SORE	m^6^A modification stabilizes SORE which activates the Wnt2b and β‐catenin	[104]
Sorafenib	Hepatocellular carcinoma (HCC)	↑RBM15B	Activation of Hippo signaling	TRAM2	Increased m^6^A on TRAM2 transcript upregulates the protein which activates the Hippo pathway	[107]
Osimertinib	Lung adenocarcinoma (LUAD)	↑METTL3	Activation of Hippo signaling	circKRT17	m^6^A‐mediated circKRT17 stabilization, nuclear YAP1 localization and transcriptional activation of resistance genes	[105]
Apatinib	Hepatocellular carcinoma (HCC)	↑METTL3	Inactivation of p53 pathway	p53	m^6^A modification causes loss of p53 function and reduces apoptosis of HCC cells	[76]
**Monoclonal antibodies**						
Cetuximab	Colorectal cancer (CRC)	↑METTL3	EV mediated resistance transfer	miR‐100 miR‐125b	METTL3‐mediated m^6^A modification of miRNAs are secreted in Evs from resistant cells transferring the resistance	[102]
Cetuximab	Colorectal cancer (CRC)	↑METTL3 ↑hnRNPA2B1	Wnt/β‐catenin pathway activation	TCF7L2	m^6^A‐modification stabilizes TCF7L2 transcript, TCF7L2 overexpression activates Wnt/β‐catenin pathway developing resistance	[37]
Anti‐PD‐L1 Anti‐CTLA‐4 monoclonal antibodies	Melanoma	↑YTHDF1	Immune evasion	Lysosomal proteins	Enhanced YTHDF1‐mediated translation of m^6^A‐modified lysosomal genes accelerates tumor antigen and MHC‐I degradation	[93]
Anti‐PD1 antibody	Melanoma	↑ALKBH5	Immune suppression	Mct4	Decreased m^6^A of Mct4 enhances its stability resulting in increased lactate secretion in TME and immune cell suppression	[97]
Pembrolizumab	Cholangiocarcinoma	↑METTL14 YTHDF2	Immune evasion	Siah2	Increased m^6^A on Siah2 transcript causes YTHDF2‐mediated degradation resulting in increased PD‐L1 expression and resistance to therapy	[91]
Nivolumab	Hepatocellular carcinoma (HCC)	↑WTAP IGF2BP3	Immune suppression	circCCAR1	m^6^A modification of circCCAR1 is stabilized by IGF2BP3 which enhances CD8^+^ T cell depletion and immune suppression	[92]
Anti‐PD1 antibody	Colorectal cancer (CRC)	↑METTL3/14	Immune suppression	STAT1	METTL3/14‐mediated m^6^A modification of STAT1 mRNA causes degradation by YTHDF1 and reduced CD8^+^ T cell infiltration	[95]
Anti‐PD1 (α‐PD1) antibody	Colorectal cancer (CRC)	↑YTHDF1	Immune suppression	p65	YTHDF1‐induced translation of m^6^A methylated p65 activates downstream molecules thereby enhancing MDSC infiltration in TME and resulting in CD8^+^ T cell inhibition	[94]
Bacillus Calmette‐Guérin (BCG) vaccine	Bladder cancer	↑YTHDF2	Immune suppression	DDX58	YTHDF2 destabilizes m^6^A‐modified DDX58 downregulating RIG‐I and thus abolishing its tumor suppressive effect	[96]
**Others**						
Olaparib Rucaparib	Epithelial ovarian cancer (EOC)	↓FTO ↓ALKBH5 ↑IGF2BP2	Wnt/β‐catenin pathway activation	FZD10	m^6^A modification stabilizes and upregulates FZD10 resulting in Wnt/β‐catenin pathway activation and PARPi resistance	[103]
**More than one classe**						
Doxorubicin Taxol	Breast cancer Liver cancer	↑METTL3	Enhanced drug efflux Enhanced fatty acid oxidation and ATP production (metabolic reprogramming)	ESRRG (ERRγ)	m^6^A‐mediated mRNA maturation and ERRγ upregulation; ERRγ‐mediated ABCB1 and Cpt1b overexpression	[59]
5‐FU Oxaliplatin	Colorectal cancer (CRC)	↑METTL3 ↑IGF2BP1	Upregulated stemness and chemoresistance‐associated genes	Sec62	Activation of Wnt/β‐catenin signaling pathway	[41]
Vincristine Doxorubicin 5‐FU	Gastric cancer	↑hnRNPA2B1	Wnt/β‐catenin pathway activation; increased stemness	lncRNA NEAT1	NEAT1 is stabilized and upregulated by hnRNPA2B1 and activates Wnt/β‐catenin signaling	[54]
Cisplatin Doxorubicin 5‐FU	Gastric cancer	METTL3	Decreased autophagy	ARHGAP5	Decreased autophagic degradation of RHGAP5‐AS1 recruits METTL3 and stabilizes RHGAP5	[68]
Doxorubicin Oxaliplatin 5‐FU	Colorectal cancer (CRC)	↑METTL3	Loss of p53 function	p53	m^6^A modification produces non‐functional p53 protein leading to multidrug resistance in CRC cells	[77]
Bortezomib (proteasome inhibitors) Melphalan (alkylating agents) Carfilzomib (proteasome inhibitors)	Multiple myeloma (MM)	↑METTL7B	Inhibition of drug‐induced apoptosis	LOC606724 SNHG1	m^6^A modified lncRNAs LOC606724 and SNHG1 are enriched in the MM‐associated adipocyte exosomes resulting in decreased cancer cell apoptosis	[79]
Trastuzumab Lapatinib	HER2‐positive Breast cancer	↑ALKBH5 YTHDF2	Metabolic alteration	GLUT4	m^6^A demethylation protects GLUT4 mRNA from YTHDF2 mediated degradation and increased glycolysis thereby promoting drug resistance	[87]

### 4.1 Effects on Drug Transport

Drug transporters are plasma membrane proteins controlling the influx or efflux of drugs inside or outside of cells. These are categorized into two main classes: the solute carrier (SLC) family and the ATP binding cassette (ABC) family. While the SLC transporters which include organic anion‐transporting polypeptides (OATPs), organic anion transporters (OATs), organic cation transporters (OCTs), peptide transporters (PEPTs) and multidrug/toxin extrusions (MATEs) are associated with drug uptake, the ABC transporters such as P‐glycoprotein (P‐GP/ABCBl), breast cancer resistance protein (BCPR/ABCG2) and multidrug resistance proteins (MRPs/ABCC1) are responsible for pumping the drugs out of the cells.^[^ ^55^ ^]^ ABC transporters manifest diverse tissue distribution notably on the membranes of enterocytes, hepatocytes, renal tubular cells, or the epithelial linings of the blood‐brain barrier (BBB). Although the primary role of ABC transporters is to protect cells from xenobiotics by effluxing them out of the cells, these transporters play important roles in causing multidrug resistance that is adopted by the cancer cells.^[^ ^56^ ^]^ Increasing evidence suggests that m^6^A modification regulators are associated with differential expression of the transporter proteins on the cancerous cells, conferring multidrug resistance (**Table** **3**). Elevated METTL3 levels were detected in imatinib‐resistant gastrointestinal stromal tumor (GIST) cells and tissue samples which insert the m^6^A modification to the 5′‐UTR of its downstream target MRP1 mRNA. Increased m^6^A in the MRP1 transcript is further recognized by the m^6^A reader YTHDF1 and eEF‐1 which enhances translation of MRP1 mRNA causing its overexpression in the cancerous cells leading to imatinib resistance due to increased drug efflux.^[^ ^57^ ^]^ METTL3 is also upregulated in ccRCC where it causes m^6^A methylation at the 5′‐UTR of the ABC transporter ABCD1 mRNA, enhancing its translation. m^6^A‐dependent upregulation of ABCD1 is associated with increased tumorigenesis and poor prognosis in ccRCC although the implication of ABCD1 in chemoresistance is not established.^[^ ^42^ ^]^ METTL3 upregulation is associated with doxorubicin resistance in breast cancer cells due to increased expression of drug efflux pumps that decrease the therapeutic concentration of the drug inside the cells. In one study, METTL3 promoted the expression of MDR1 and ABCG2 (commonly known as breast cancer‐resistant protein, BCRP) through m^6^A modification and maturation of miR‐221‐3p.^[^ ^58^ ^]^ In chemoresistant breast and liver cancer, increased expression of METTL3 indirectly causes resistance of cancerous cells to doxorubicin and taxol. METTL3‐mediated m^6^A methylation of the ESRRG mRNA enhances its splicing and maturation which translates to estrogen receptor‐related receptor‐γ (ERRγ) causing its upregulation. ERRγ along with p65 subsequently binds to the promoter region of ABCB1 increasing its transcription. Increased expression of ABCB1 (P‐gp), a drug efflux pump confers the cancer cells' resistance to doxorubicin and taxol via decreased intracellular drug concentration.^[^ ^59^ ^]^ Another m^6^A writer, WTAP, produces cisplatin resistance in NK/T cell lymphoma (NKTCL) via m^6^A‐mediated upregulation of DUSP6. Increased DUSP6 induces the expression of drug efflux proteins MRP1 and P‐gp, contributing to cisplatin resistance.^[^ ^60^ ^]^ Increased ABCB1 expression and Adriamycin (doxorubicin) resistance due to direct m^6^A modification of the ABCB1 mRNA was also observed in breast cancer cells. Doxorubicin resistance in the cancerous cells is due to the upregulation of lncRNA A1BG‐AS1 which recruits the m^6^A reader IGF2BP2 to the m^6^A modification sites on ABCB1 mRNA causing its stabilization and translational upregulation. m^6^A‐mediated drug efflux pump ABCB1 overexpression thus causes doxorubicin resistance in breast cancer cells although which writer is causing the modification was not examined in this study.^[^ ^61^ ^]^ An indirect regulatory role for m^6^A modification and its reader YTHDF2 on ABCB1 expression was also observed in tamoxifen‐resistant breast cancer cells. In this case, decreased m^6^A methylation at the 5′‐UTR region of the transcription factor ATF3 mRNA and YTHDF2 downregulation in the breast cancer cells enhances the stabilization and translational efficiency of ATF3 transcripts. ATF3 elevation, in turn, stimulates increased expression of ABCB1 efflux pumps resulting in enhanced tamoxifen efflux conferring the breast cancer cells' resistance to this drug.^[^ ^62^ ^]^

**Table 3:** Drug transporter expression.

Cancer Type	Drug Transporter	Writer/ Eraser	m^6^A Reader	Effect on Transcript	Reference
Gastrointestinal stromal tumor (GIST)	MRP1	↑METTL3	YTHDF1 eEF‐1	m^6^A enrichment at 5′‐UTR; increased MRP1 translation by YTHDF1	[57]
Clear cell renal cell carcinoma (ccRCC)	ABCD1	↑METTL3	Not involved	m^6^A enrichment at 5′‐UTR; cap‐independent translation	[42]
Breast cancer Liver cancer	ABCB1 (P‐gp)	↑METTL3	Unknown	Increased m^6^A in ESRRG mRNA enhances splicing and ERRγ expression which upregulates ABCB1	[59]
Non‐small cell lung cancer	ABCC10	↑FTO	YTHDF2	Decreased m^6^A at 5′‐UTR of ABCC10 mRNA; decreased mRNA decay causes ABCC1 overexpression	[64]
Breast cancer	MDR1 BCRP (ABCG2)	↑METTL3	Unknown	m^6^A modification promotes miR‐221‐3p maturation which upregulates MDR1 and BCRP (ABCG2)	[58]
Breast cancer	ABCB1	Unknown	IGF2BP2	Enhanced IGF2BP2 m^6^A binding on ABCB1 mRNA and transcript stabilization	[61]
Breast cancer	ABCB1	Unknown	↓YTHDF2	Decreased m^6^A on ATF3 mRNA and YTHDF2 downregulation enhances ATF3 stabilization; ATF3 enhances transcriptional activation and upregulation of ABCB1	[62]
Colorectal cancer (CRC)	ABCB1	Unknown	↑IGF2BP3	IGF2BP3 binds to m^6^A on ABCB1 mRNA promoting its stability and upregulation	[63]
Colorectal cancer (CRC)	ATP7A	Unknown	↑YTHDF3	ATP7A overexpression resulting in efflux of platinum drugs	[65]
Hepatocellular carcinoma (HCC)	OATP1B1 OATP1B3	↓METTL14	IGF2BPs	Lower METTL14 expression decreases m^6^A on HNF3γ mRNA with lower downstream expression of drug transporters	[30]
Hepatocellular carcinoma (HCC)	OATP1B1 OATP1B3	METTL3	IGF2BP1	Increased m^6^A on NIFK‐AS1 increases its stability which downregulates the drug transporters	[39]

In CRC, m^6^A‐mediated ABCB1 upregulation conferred doxorubicin resistance to the cancer cells. The m^6^A reader IGF2BP3 is upregulated in these cells which directly binds with ABCB1 mRNA, promoting its stabilization and overexpression and leading to increased doxorubicin efflux.^[^ ^63^ ^]^ The m^6^A eraser FTO is upregulated in metastatic non‐small cell lung cancer (NSCLC), which is associated with overexpression of drug transporter ABCC10. FTO upregulation decreases m^6^A abundance in ABCC10 transcript which otherwise would be recognized by the m^6^A reader YTHDF2 and cause its decay. This reduction in m^6^A in ABCC10 mRNA enhances its stability and upregulation and results in efflux of gefitinib which is a substrate of ABCC10 and confers the NSCLCs gefitinib resistance.^[^ ^64^ ^]^ Not only the overexpression of drug efflux pumps contributes to chemotherapeutic resistance in cancer cells, downregulation of drug uptake transporters also plays a role in resistance development as in the case of hepatocellular carcinoma cells.^[^ ^30^ ^]^ HNF3γ downregulation due to METTL14 downregulation in poorly differentiated HCC cells or CSCs also causes decreased expression of sorafenib transporters OATP1B1 and OATP1B3 for which HNF3γ acts as the transcriptional activator of these transporter proteins. Lower drug transporter expression also decreases drug concentration inside the poorly differentiated cancer cells and CSCs, resulting in poor sorafenib response.^[^ ^30^ ^]^ Chen et al. on the other hand revealed a different pathway for sorafenib resistance in HCC which involves NIFK‐AS1‐mediated downregulation of OATP1B1 and OATP1B3. They showed that METTL3‐mediated increase of m^6^A methylation and upregulation of the lncRNA NIFK‐AS1 resulted in lower OATP1B1 and OATP1B3 expression which led to reduced sorafenib uptake.^[^ ^39^ ^]^ In colorectal cancer, increased YTHDF3 expression correlates with oxaliplatin resistance via enhanced expression of platinum drug transporters. YTHDF3 upregulation recognizes m^6^A at the 5′‐UTR of the copper efflux transporters, ATP7A and DYRK1B; these are positive regulators of Hedgehog (HH)/GLI signaling pathway and recruit eIF3A which causes their upregulation. Increased expression of these proteins results in oxaliplatin resistance in CRC cells.^[^ ^65^ ^]^

### 4.2 Modulation of Autophagy

Autophagy is a process of self‐devouring among cells in which the organelles of some cells are recycled to be utilized as sources of essential macromolecular building blocks for surviving cells in response to stressors such as nutrient deprivation or hypoxia. Modulation of this process is seen in advanced stages of cancers where it is exploited by established tumor cells for their survival.^[^ ^66^ ^]^ In the early stages, this may be a mechanism for the destruction of cancer cells by chemotherapeutic agents; however, it is a highly plastic or dynamic mechanism to support the survival of chemoresistant cells.^[^ ^67^ ^]^ m^6^A‐mediated modulation of autophagy also contributes to the development of resistance to different therapeutic agents (**Table** **4**). Impaired autophagic degradation and overexpression of lncRNA ARHGAP5‐AS1 was observed in gastric cancer, which recruits METTL3 for m^6^A‐mediated stabilization of ARHGAP5 mRNA rendering cancer cells resistant to doxorubicin, cisplatin and 5‐FU.^[^ ^68^ ^]^ Another lncRNA, LINC00470, recruits METTL3 and increases m^6^A modification on PTEN mRNA resulting in PTEN degradation in AML. PTEN downregulation subsequently inhibits autophagy, resulting in resistance to doxorubicin.^[^ ^58^ ^]^ METTL3‐mediated autophagy was also observed in non‐small cell lung cancer resulting in gefitinib resistance. Through m^6^A modification, METTL3 upregulates autophagy‐related LC3, ATG5, and ATG7 expression which contribute to resistance development to gefitinib.^[^ ^69^ ^]^ In contrast, METTL3 downregulation promotes sorafenib resistance and activates autophagic pathways in hepatocellular carcinoma. METTL3 depletion decreases m^6^A levels in FOXO3 mRNA at the 3′‐UTR region and downregulates it; otherwise, FOXO3 mRNA would be read by YTHDF1 stabilizing the transcript. FOXO3 downregulation thus activates autophagic pathways and generates sorafenib resistance in HCC cells.^[^ ^70^ ^]^ METTL16 downregulation is associated with cisplatin resistance in bladder cancer in which hypoxia‐inducible factor 2α (HIF‐2α) represses transcription of METTL16. Suppression of METTL16 decreases m^6^A on the prostate transmembrane protein, androgen induced‐1 (PMEPA1), a regulator of autophagy, and reduces its stability. This contributes to bladder cancer cisplatin resistance due to suppression of autophagy.^[^ ^71^ ^]^ Decreased m^6^A level due to FTO overexpression also contributes to cisplatin resistance in gastric cancer through the induction of autophagy. FTO demethylates Unc‐51‐like kinase 1 (ULK‐1) protecting it from YTHDF2‐mediated degradation and ULK‐1 contributes to cisplatin resistance via modulation of autophagy.^[^ ^72^ ^]^

**Table 4:** Modulation of autophagy.

Cancer type	Molecular target of autophagy	Writer/ Eraser	m^6^A Reader	Effect on Transcript	Reference
Gastric cancer	ARHGAP5‐AS1	↑METTL3	HuR	Decreased autophagy of ARHGAP5‐AS1 causes m^6^A enrichment on ARHGAP5 and increased chemoresistance	[68]
Gastric cancer	ULK1	↑FTO	YTHDF2	FTO‐mediated ULK1 demethylation protects the transcript from degradation which modulates autophagy	[72]
Acute myeloid leukemia (AML)	PTEN	↑METTL3	Unknown	Increased m^6^A of PTEN mRNA results in its degradation which upregulates genes that inhibit autophagy and chemoresistance	[58]
Non‐small cell lung cancer (NSCLC)	LC3, ATG5ATG7	↑METTL3	Unknown	m^6^A‐mediated upregulation of autophagy‐associated genes	[69]
Hepatocellular carcinoma (HCC)	FOXO3	↓METTL3	YTHDF1	Decreased m^6^A at 3′‐UTR of FOXO3 decreases its YTHDF1‐mediated stabilization activating autophagy	[70]
Bladder cancer	PMEPA1	↓METTL16	No	Low METTL16 expression decreases m^6^A‐mediated PEMPA1 stability, decreasing autophagy	[71]

### 4.3 Modulation of Apoptotic Gene Expression

METTL3 overexpression due to decreased lncRNA MEG3 reduces arabinocytosine (AraC)‐induced apoptosis in acute myeloid leukemia cells. METTL3‐mediated m^6^A modification causes MYC upregulation and is responsible for the AraC resistance of the AML cells.^[^ ^73^ ^]^ m^6^A plays a role in cisplatin resistance in gastric^[^ ^46^, ^74^ ^]^ and bladder cancer^[^ ^75^ ^]^ cells due to alteration in the apoptotic process. Circular RNA circ0008399 and WTAP are upregulated in bladder cancer which causes m^6^A‐mediated alterations of apoptosis‐associated genes. circ0008399 promotes METTL3/METTL14/WTAP complex formation that, in turn, increases m^6^A levels in TNF alpha‐induced protein3 (TNFAIP3) mRNA thus enhancing its stability and expression. TNFAIP3 overexpression represses apoptosis of bladder cancer cells causing cisplatin resistance.^[^ ^75^ ^]^ In gastric cancer, increased lncRNA LINC00942 resulted in decreased ubiquitination of the RNA‐binding protein MSI2. In cancer cells, m^6^A writer complex (METTL3/METTL14/WTAP)‐mediated modification of c‐Myc mRNA is protected from YTHDF2‐mediated degradation by MSI2 with c‐Myc overexpression. Increased c‐Myc levels are associated with stemness and decreased cisplatin‐induced cell apoptosis, thus inducing resistance to cisplatin.^[^ ^46^ ^]^ However, in another study involving gastric cancer patients demonstrated that METTL3‐mediated m^6^A modification and YTHDC1‐induced stabilization of FAM120A mRNA increases FAM120A expression which inhibits cancer cell ferroptosis and is responsible for cisplatin resistance.^[^ ^74^ ^]^ METTL3‐catalyzed m^6^A modification inactivates p53 and decreases the sensitivity of HCC cells against apatinib treatment through modulation of p53‐regulated apoptotic protein expression.^[^ ^76^ ^]^ Similarly, Uddin et al. showed that an m^6^A modification in p53 pre‐mRNA results in mutant protein expression in colorectal cancer cells which convert chemosensitive cells to be resistant to multiple chemotherapeutic agents including doxorubicin, oxaliplatin, and 5‐FU.^[^ ^77^ ^]^ Another m^6^A writer METTL14 is upregulated in renal cell carcinoma which contributes to sunitinib resistance through suppression of apoptosis. METTL14 increases m^6^A modification in TNF receptor‐associated factor 1 (TRAF1) which is recognized and stabilized by IGF2BP2 causing its upregulation. TRAF1 upregulation induces sunitinib resistance by suppressing angiogenic and apoptotic protein expression.^[^ ^78^ ^]^ WTAP upregulation suppresses cisplatin‐induced apoptosis of NK/T cell lymphoma (NKTCL) via DUSP6 overexpression. WTAP‐mediated m^6^A modification of DUSP6 mRNA induces its expression which further increases the expression of antiapoptotic protein Bcl‐2 and decreases proapoptotic protein BAX.^[^ ^60^ ^]^ A newly discovered m^6^A writer METTL7B decreases apoptosis of multiple myeloma (MM) cells through enrichment of lncRNAs LOC606724 and SNHG1 in exosomes of MM‐associated adipocytes in the tumor microenvironment. The m^6^A methylated LOC606724 and SNHG1 are enriched in adipocyte exosomes that protect the MM cells from bortezomib, melphalan, and carfilzomib‐induced apoptosis and contribute to resistance to these drugs.^[^ ^79^ ^]^ Contrary to previous studies, ALKBH5‐mediated m^6^A demethylation is associated with dexamethasone resistance in T cell acute lymphoblastic leukemia (T‐ALL). ALKBH5, by reducing m^6^A, enhances ubiquitin‐specific protease 1 (USP1) expression that regulates chemoresistance by upregulating Aurora B.^[^ ^80^ ^]^ Thus, m^6^A modification regulates apoptosis in cancer therapy resistance (**Table** **5**).

**Table 5:** Decreased apoptosis.

Cancer type	Molecular target of apoptosis	Writer/Eraser	m^6^A Reader	Effect on Transcript	Reference
Acute myeloid leukemia (AML)	MYC	METTL3	Unknown	METTL3‐mediated m^6^A modification increases MYC expression leading to decreased apoptosis	[73]
Hepatocellular carcinoma (HCC)	p53	METTL3	Unknown	Inactivation of p53 due to m^6^A modification	[76]
Breast cancer	Bcl‐2	WTAP	YTHDF1	Increased Bcl‐2 translation due to m^6^A modification and reduced apoptosis	[123]
Gastric cancer	c‐Myc	METTL3‐METTL14‐WTAP	Inhibition of YTHDF2	m^6^A modification of c‐Myc mRNA stabilization decreases cisplatin‐induced cancer cell apoptosis	[46]
Gastric cancer	FAM120A	METTL3	YTHDC1	m^6^A on FAM120A is recognized and stabilized by YTHDC1. FAM120A overexpression inhibits ferroptosis	[74]
Bladder cancer	TNFAIP3	METTL3‐METTL14‐WTAP	Unknown	Increased m^6^A causes TNFAIP3 stabilization and overexpression suppresses apoptosis	[75]
Colorectal cancer (CRC)	p53	METTL3	Unknown	m^6^A modification of p53 pre‐mRNA leads to mutant protein expression and results in decreased apoptosis	[77]
Renal cell carcinoma (RCC)	TRAF1	METTL14	IGF2BP2	m^6^A modification enhances TRAF1 stabilization resulting in suppression of apoptosis	[78]
Multiple myeloma (MM)	LOC606724 SNHG1	METTL7B	Unknown	m^6^A modified LOC606724 and SNHG1 enrichment in adipocyte‐derived exosomes inhibit apoptotic cancer cell death	[79]
T cell acute lymphoblastic leukemia (T‐ALL)	USP1	ALKBH5	Unknown	m^6^A demethylation of USP1 by ALKBH5 increases its expression reducing cell apoptosis	[80]

### 4.4 Induction of DNA Damage Repair

Direct DNA strand break or DNA damage are mechanisms by which chemotherapeutic agents bring about cancer cell death. Resistance of cancer cells to these drugs occurs due to rapid repair of DNA lesions and m^6^A modifications and its regulators are associated with this process (**Table** **6**). In gastric cancer stem‐like cells, METTL3 upregulation is responsible for PARP1 overexpression which is associated with oxaliplatin resistance due to DNA damage repair. In gastric cancer cells, METTL3 introduces m^6^A to the 3′‐UTR region of the PARP1 mRNA where the reader YTHDF1 is recruited; this increases its stability and expression. PARP1, in turn, initiates the base excision repair pathway and repairs DNA damage caused by oxaliplatin leading to oxaliplatin resistance.^[^ ^81^ ^]^ In seminoma, resistance to another platinum drug, cisplatin is due to m^6^A‐dependent overexpression of DNA damage repair genes. METTL3 upregulation in seminoma cells increases m^6^A in the TFAP2C transcript which is stabilized by IGF2BP1. TFAP2C in turn activates the DNA repair genes WEE1 G2 checkpoint kinase (WEE1) and breast cancer type 1 (BRCA1) attenuating cisplatin‐induced DNA damage.^[^ ^82^ ^]^ METTL3‐mediated m^6^A modification is an underlying cause of temozolomide resistance in glioblastoma due to DNA damage repair. In glioma cancer cells, METTL3 expression is upregulated causing increased m^6^A methylation of O^6^‐methylguanine‐DNA methyltransferase (MGMT) and alkylpurine‐DNA‐N‐glycosylase (APNG) mRNAs leading to their overexpression. MGMT and APNG are the major DNA damage repair enzymes that repair lesions caused by temozolomide leading to resistance to this drug.^[^ ^83^ ^]^ The m^6^A reader YTHDC1 plays a vital role in DNA damage repair and Adriamycin (doxorubicin) resistance in breast cancer cells, the expression of which is suppressed by epithelial membrane protein 3 (EMP3). EMP3 overexpression inhibits breast cancer cell proliferation, overcoming chemoresistance, and is associated with more favorable patient survival.^[^ ^84^ ^]^ Likewise, doxorubicin resistance in triple‐negative breast cancer (TNBC) is due to low reactive oxygen species (ROS) and superoxide dismutase (SOD2) overexpression caused by FOXO1, a master regulator of cellular ROS. Here, overexpression of ALKBH5 demethylates FOXO1 mRNA and increases its expression leading to CSCs promotion and doxorubicin resistance.^[^ ^85^ ^]^

**Table 6:** DNA damage repair.

Cancer type	Molecular target of m^6^A modification	Writer/ Eraser	m^6^A Reader	Effect on Transcript	Reference
Gastric cancer	PARP1	↑METTL3	YTHDF1	Increased m^6^A stabilizes the PARP1 transcript which initiates DNA damage repair	[68]
Seminoma	TFAP2C	↑METTL3	IGF2BP1	m^6^A‐rich TFAP2C transcript is stabilized by IGF2BP1 which in turn activates DNA damage repair genes	[82]
Glioblastoma	MGMT APNG	↑METTL3	Unknown	Increased m^6^A on target transcripts enhances mRNA expression	[83]
Breast cancer	BRCA1/RAD51	↑METTL3	↑YTHDC1	YTHDC1 enhances BRCA1/RAD51 expression	[84]
TNBC	FOXO1	↑ALKBH5	Not involved	ALKBH5‐mediated demethylation and increased expression of FOXO1 (cellular ROX regulator)	[85]

### 4.5 Metabolic Reprogramming

Metabolic alteration is frequently observed in cancer cells; this also contributes to the development of resistance to anticancer drugs. m^6^A regulators through insertion or removal of this modification to the molecules in metabolic pathways contribute to cancer therapy resistance (**Table** **7**). METTL3 upregulation is associated with chemoresistance in breast and liver cancer due to metabolic reprogramming inside the cells. Increased m^6^A methylation by METTL3 to its target ESRRG transcript enhances mRNA maturation and translational expression of ERRγ. ERRγ, upon complex formation with p65, binds to the promoter region of CPT1B and increases expression of Cpt1b which in turn is responsible for mitochondrial fatty acid oxidation (FAO) and enhanced ATP generation and oxygen consumption rate (OCR) in cancer cells.^[^ ^59^ ^]^ FTO‐mediated m^6^A removal and stabilization of target mRNA transcript produces temozolomide resistance in glioblastoma. lncRNA JPX recruits FTO on phosphoinositide‐dependent kinase‐1 (PDK1), which demethylates the transcript and protects it from degradation, resulting in enhanced glucose uptake and lactate production leading to temozolomide resistance.^[^ ^86^ ^]^ Increased glycolysis due to GLUT4 overexpression is responsible for therapeutic resistance in HER2‐positive breast cancer. In breast cancer cells ALKBH5 overexpression erases m^6^A methylation from the GLUT4 mRNA, protecting it from YTHDF2‐mediated degradation and increases its expression through stabilizing the transcript. GLUT4 overexpression enhances glycolysis in HER2‐positive breast cancer cells resulting in resistance to HER2‐targeting trastuzumab and lapatinib.^[^ ^87^ ^]^ In cisplatin‐resistant colon cancer cells, increased YTHDF1 is responsible for cisplatin resistance due to alteration in glutamine metabolism. In resistant CRC cells, YTHDF1 recognizes m^6^A at the 3′‐UTR region of the glutaminase 1 (GLS1) mRNA which promotes GLS1 protein synthesis. GLS1 upregulation enhances glutamine uptake and metabolism in the cancer cells leading to cisplatin resistance.^[^ ^88^ ^]^

**Table 7:** Metabolic reprogramming.

Cancer Type	Metabolic Alteration	Writer/Eraser	m^6^A Reader	Effect on Transcript	Reference
Breast cancer Liver cancer	↑Mitochondrial fatty acid oxidation (FAO) ↑Oxygen consumption rate (OCR)	↑METTL3	Unknown	Increased m^6^A in ESRRG mRNA enhances splicing and ERRγ expression which upregulates CPT1B	[59]
Colorectal cancer (CRC)	↑Glutamine metabolism	Unknown	↑YTHDF1	YTHDF1/m^6^A‐mediated GLS1 overexpression and increased glutamine uptake and metabolism	[88]
Glioblastoma	↑Glucose uptake and lactate production	↑FTO	Unknown	FTO‐mediated demethylation and stabilization of PDK1 resulting in increased aerobic glycolysis	[86]
Breast cancer (HER2‐positive)	↑Glycolysis	↑ALKBH5	YTHDF2	ALKBH5 mediated GLUT4 mRNA demethylation protects it from YTHDF2 mediated degradation	[87]

### 4.6 Tumor Microenvironment (TME) and Immune Evasion

Immune evasion is a hallmark of cancer which is exploited by cancer cells for their survival and expansion.^[^ ^89^ ^]^ Another method of resistance to therapy for cancer cells is to manipulate immune cells and checkpoints through m^6^A modification (**Table** **8**). METTL3 contributes to the immune escape of bladder cancer cells through PD‐L1 overexpression. m^6^A modification of PD‐L1 mRNA is recognized by IGF2BP1 which stabilizes the transcript, increasing PD‐L1 expression in bladder cancer cells.^[^ ^90^ ^]^ METTL14‐induced ICI resistance occurs in cholangiocarcinoma with increased expression of PD‐L1 in cancer cells. METTL14 enhanced m^6^A methylation on the seven in absentia homolog 2 (Siah2) ubiquitin ligase which is responsible for proteasomal degradation of PD‐L1 in cancer cells. m^6^A‐modified Siah2 transcript is degraded by YTHDF2; this results in the upregulation of PD‐L1 in the cancer cell surface leading to an altered response to pembrolizumab.^[^ ^91^ ^]^ WTAP‐mediated m^6^A modification also contributes to increased anti‐PD‐1 therapy resistance in HCC cells. m^6^A methylation on the circular RNA circCCAR1 enhances its stability through IGF2BP3 that is secreted in the exosomes of the cancer cells. circCCAR1 is taken up by CD8^+^ T cells causing enhanced PD‐1 expression and results 121 in CD8^+^ T cell exhaustion, thereby rendering the cells resistant to nivolumab (Opdivo).^[^ ^92^ ^]^ Increased expression of the m^6^A reader YTHDF1 is implicated in tumor immunity against immune checkpoint inhibitors in melanoma and CRC. In melanoma, YTHDF1 enhances lysosomal gene translation and protein expression thereby enhancing lysosome generation. Lysosomal proteolytic digestion is responsible for immune evasion of cancer cells by degradation of tumor‐specific antigens and MHC‐I resulting in resistance to anti‐PD‐L1 and anti‐CTLA‐4 monoclonal antibodies.^[^ ^93^ ^]^ In CRC, YTHDF1 enhances translation of the m^6^A‐modified p65 which upregulates CXCL1. CXCL1 increases infiltration of myeloid‐derived suppressor cells (MDSCs) in the tumor microenvironment resulting in CD8^+^ T cell suppression and developing resistance to anti‐PD1 (α‐PD1) therapy.^[^ ^94^ ^]^ In a cohort of CRC patients, METTL3/METTL14 catalyzed m^6^A modification of STAT1 suppressed signaling through YTHDF2‐mediated degradation of STAT1 transcript, rendering the cells resistant to anti‐PD1 antibody therapy; loss of METTL3/METTL14 sensitizes them.^[^ ^95^ ^]^ Another m^6^A reader, YTHDF2, is responsible for the decreased antitumor efficacy of Bacillus Calmette‐Guérin (BCG) vaccine immunotherapy treatment in urothelial bladder cancer. YTHDF2 destabilizes DDX58 mRNA, which encodes for RIG‐I; this results in reduced CD8^+^ T lymphocyte infiltration into the tumor tissues and the urothelium.^[^ ^96^ ^]^ The m^6^A demethylase, ALKBH5, contributes to anti‐PD1 antibody therapy resistance in melanoma by enhancing the stability of its target transcript Mct4 which otherwise would be degraded by YTHDF2. Enhanced Mct4 is necessary for lactate secretion in the tumor microenvironment resulting in immune suppression through reduced infiltration of T cells.^[^ ^97^ ^]^

**Table 8:** TME/Immune evasion.

Cancer Type	Immune/TME cell/Molecular Target	Writer/Eraser	m^6^A Reader	Effect on Transcript	Reference
Bladder cancer	↓CD8^+^ T cell/PD‐L1	↑METTL3	↑IGF2BP1	m^6^A modification of PD‐L1 mRNA increases its stability and expression	[90]
Breast cancer Liver cancer	↑M2‐Macrophage	↑METTL3	Unknown	Increased m^6^A in TRAF5 mRNA enhances its degradation	[98]
Triple negative breast cancer (TNBC)	↑Mφ‐Macrophage (Pro‐tumoral)	Unknown	↑YTHDF2	Unknown	[99]
Colorectal cancer (CRC)	↓CD8^+^ T cell/STAT1	↑METTL3/14	↑YTHDF2	Increased m^6^A on STAT1 mRNA enhances its degradation	[95]
Melanoma	Cancer cell lysosome proteins	Unknown	↑YTHDF1	YTHDF1‐mediated translation of m^6^A‐modified lysosomal transcripts upregulates lysosomal protein expression	[93]
Cholangiocarcinoma	Siah2	↑METTL14	↑YTHDF2	m^6^A‐modified Siah2 transcript degradation by YTHDF2 increases PD‐L1 expression	[91]
Hepatocellular carcinoma (HCC)	↓CD8^+^ T cell/circCCAR1	↑WTAP	IGF2BP3	m^6^A‐modified circCCAR1 stabilization and exosomal excretion resulting in CD8^+^ T cell depletion	[92]
Melanoma	↓Tregs and MDSC/Mct4	↑ALKBH5	Not involved	m^6^A reduction on Mct4 transcript protects it from degradation which increases lactate secretion and immune cell suppression	[97]
Colorectal cancer (CRC)	↑MDSC ↓CD8^+^ T cell	Unknown	↑YTHDF1	YTHDF1 upregulates p65 which enhances MDSC migration through activation of downstream targets; this results in anti‐PD1 resistance via suppression of CD8^+^ T cells	[94]
Bladder cancer	↓RIG‐I ↓CD8^+^ T cell	Unknown	↑YTHDF2	m^6^A‐modified DDX58 degradation by YTHDF2 downregulates RIG‐I resulting in decreased CD8^+^ T cells in tumor	[96]
Breast cancer	↓CD8^+^ T cell/TFAP2A	↑VIRMA	↑HNRNPC	m^6^A modification increase s TFAP2A expression which enhances DDR1 transcription	[100]

An immunosuppressive microenvironment also contributes to chemoresistance via elevated m^6^A levels in cancer cells. Increased infiltration of M2‐type macrophages in CRC cells induces METTL3 expression which enhances m^6^A levels in the TRAF5 transcript (an inducer of necroptosis) causing its destabilization and downregulation and resulting in oxaliplatin‐induced necroptosis inhibition and resistance.^[^ ^98^ ^]^ Protumorigenic polarization of the macrophage population was also observed in breast cancer samples from triple‐negative breast cancer (TNBC) patients receiving chemotherapy. These protumorigenic macrophages show increased expression of the m^6^A reader, YTHDF2, although it is not clear how YTHDF2 contributes to protumoral macrophage polarization and chemoresistance.^[^ ^99^ ^]^ VIRMA‐mediated m^6^A modification also promotes the immune escape of metastatic breast cancer cells by promoting collagen alignment in the extracellular matrix (ECM) and reducing CD8^+^ T cell infiltration in the tumor microenvironment. m^6^A modification of transcription factor AP‐2 A (TFAP2A) mRNA is enhanced by HNRNPC resulting in its upregulation. TFAP2A further binds to the discoidin domain receptor 1 (DDR1) promoter and upregulates it, resulting in ECM collagen alignment and prevention of CD8^+^ T cell infiltration.^[^ ^100^ ^]^

### 4.7 Other Mechanisms

#### 4.7.1 Enzymatic Inactivation

After the drug reaches the site of action, metabolic inactivation of the molecule may lead to therapeutic failure. m^6^A‐mediated drug‐metabolizing enzyme induction may play important roles in the development of therapeutic resistance to cancer cells. This mode of chemoresistance occurs in gemcitabine‐resistant pancreatic cancer cells. METTL14 is upregulated in gemcitabine‐resistant cancer cells which increases the expression of gemcitabine metabolizing enzyme cytidine deaminase (CDA). Increased CDA enhances metabolic deactivation of gemcitabine leading to resistance in pancreatic cancer cells.^[^ ^101^ ^]^

#### 4.7.2 Extracellular Vesicle Mediated

METTL3 controls extracellular vesicle (EV)‐mediated transfer of drug resistance to non‐resistant cancer cells. METTL3‐mediated m^6^A modification of microRNAs miR‐100 and miR‐125b are enriched in EVs secreted from the cetuximab‐resistant CRC cells, which upon exposure to non‐resistant cancer cells exhibit resistance to cetuximab.^[^ ^102^ ^]^

## 5 Cellular Signaling Pathways Underlying Chemoresistance

Oncogenic signal transduction pathways are activated in different cancer types, targeting of which is a common strategy for anticancer drug development. However, cancer cells adopt alternative signaling pathways to shun the toxic effects of these signaling molecules inhibitors and acquire resistance. m^6^A methylation contributes to aberrantly activate signaling processes and to resistance development in this way (**Table** **9**). METTL3 overexpression is responsible for multidrug resistance in CRC cells. Increased m^6^A methylation of the Sec62 mRNA, which is a regulator of stemness in CRC cells, is recognized by IGF2BP1 and enhances its stability. Sec62 upregulation further activates the Wnt/β‐catenin pathway via binding Sec62 to β‐catenin, disrupting the interaction between β‐catenin and APC, thus inhibiting β‐catenin degradation. Activation of Wnt/β‐catenin signaling pathway enhances CRC cell stemness and renders them resistant to 5‐flurouracil (5‐FU) and oxaliplatin.^[^ ^41^ ^]^ In addition, in CRC drug resistance, METTL3‐mediated m^6^A modification and hnRNPA2B1 reading are involved in cetuximab (an anti‐EGFR monoclonal antibody) resistance through activation of Wnt/β‐catenin signaling. m^6^A‐modified TCF7L2 binds both to hnRNPA2B1 and ncRNA host gene MIR100HG which stabilizes and upregulates TCF7L2. TCF7L2 activates Wnt/β‐catenin signaling resulting in cetuximab resistance.^[^ ^37^ ^]^ Multidrug resistance to vincristine, doxorubicin, and 5‐FU due to hnRNPA2B1 overexpression and Wnt/β‐catenin pathway activation occurs in gastric cancer cells. hnRNPA2B1 does so by recognition of m^6^A on lncRNA NEAT1, stabilizing it, and causing its upregulation which in turn activates the Wnt/β‐catenin signaling.^[^ ^54^ ^]^ Resistance to PARP inhibitors (PARPi), olaparib and rucaparib, is associated with the m^6^A modification‐regulated Wnt/β‐catenin pathway activation in epithelial ovarian carcinoma (EOC). In EOC cells, downregulation of FTO and ALKBH5 increases m^6^A levels on FZD10 mRNA which is read by IGF2BP2 resulting in its stabilization and upregulation. FZD10 overexpression subsequently activates the Wnt/β‐catenin signaling rendering the cells PARPi resistance.^[^ ^103^ ^]^ Wnt/β‐catenin signaling pathway activation and sorafenib resistance have also been reported in hepatocellular carcinoma. In HCC cells, m^6^A modification of the circular RNA SORE by methyltransferase complex (MTC) increases its stability and upregulation of circ‐RNA SORE which activates Wnt2b and β‐catenin thereby inducing sorafenib resistance.^[^ ^104^ ^]^ METTL3 upregulation in Gefitinib and Osimertinib‐resistant lung adenocarcinoma (LUAD) mediates resistance by YAP/TAZ (Hippo) signaling pathway activation. METTL3 incorporates m^6^A to circKRT17, enhancing its stabilization which in turn promotes YAP1 nuclear localization and leads to elevated expression of resistance‐related proteins P‐gp and MRP‐1 and survival‐associated proteins c‐Myc, cyclin‐D1, and Bcl2.^[^ ^105^ ^]^ Alternatively, METTL3 indirectly represses LATS2 (a component of Hippo pathway) through upregulation of lncRNA SNHG17 by m^6^A modification.^[^ ^106^ ^]^ Activation of the Hippo signaling pathway is also associated with sorafenib resistance in hepatocellular carcinoma which is mediated by RBM15B. In HCC cells, RBM15B is upregulated, incorporating m^6^A modification to the 3′‐UTR of TRAM2; this is associated with Hippo signaling. m^6^A‐mediated TRAM2 upregulation contributes to the sorafenib resistance of HCC.^[^ ^107^ ^]^

**Table 9:** Cellular signaling pathway associated with chemoresistance.

Cancer Type	Signaling Pathway	Writer/Eraser	m^6^A Reader	Effect on Transcript	Reference
Glioma stem cell (GSC)	Wnt/β‐catenin	↑METTL3	↑YTHDF2	m^6^A modification of LINC00839 is read and stabilized by YTHDF2 which further activates Wnt/β‐catenin signaling	[120]
Colorectal cancer (CRC)	Wnt/β‐catenin	↑METTL3	↑IGF2BP1	m^6^A enrichment on Sce62 mRNA; decreased β‐catenin degradation by Sec62; enhanced transcription of stemness and resistant genes	[41]
Colorectal cancer (CRC)	Wnt/β‐catenin	↑METTL3	↑hnRNPA2B1	m^6^A modified TCF7L2 transcript is stabilized by hnRNPA2B1 which activates Wnt signaling	[37]
Gastric cancer (GC)	Wnt/β‐catenin	Unknown	↑hnRNPA2B1	hnRNPA2B1 stabilizes m^6^A modified lncRNA NEAT1 which activates Wnt/β‐catenin signaling	[54]
Hepatocellular carcinoma (HCC)	Wnt/β‐catenin	MTC	Unknown	m^6^A methylation stabilizes circRNA‐SORE which upregulates Wnt2b and β‐catenin	[104]
Colorectal cancer (CRC)	Hedgehog (HH)/GLI	Unknown	↑YTHDF3	DYRK1B overexpression resulting in activation of Hedgehog signaling	[65]
Ovarian cancer	PI3K/Akt	↑METTL3	↑IGF2BP1	m^6^A modification stabilizes circular RNA circPLPP4 which upregulates PIK3R1	[39, 109]
Bladder cancer	PI3K/Akt	Unknown	↓YTHDC1	YTHDC1 downregulation destabilizes PTEN which activates PI3K/Akt pathway	[110]
Epithelial ovarian cancer (EOC)	JAK2/STAT3 signaling	↑ALKBH5	Not involved	ALKBH5‐mediated demethylation protects JAK2 mRNA from degradation	[112]
Breast cancer	STAT3 activation	↑FTO	Not involved	FTO‐mediated decreased m^6^A on STAT3 induces its activation	[58]
Epithelial ovarian cancer (EOC)	Wnt/β‐catenin	↓FTO ↓ALKBH5	↑IGF2BP2	Increased m^6^A on FZD10 transcript causes FZD10 stabilization and upregulation which activates the Wnt signaling	[103]
Pancreatic ductal adenocarcinoma (PDAC)	Wnt signaling	↓ALKBH5	Not involved	ALKBH downregulation decreases WIF1 and its inhibition of Wnt signaling	[113]
Hepatocellular carcinoma (HCC)	MAPK/ERK pathway	↑METTL3	Unknown	METTL3 incorporates m^6^A on DUXAP8 and upregulates it which activates MAPK1	[114]
Hepatocellular carcinoma (HCC)	Hippo pathway	↑RBM15B	Unknown	RBM15B increases m^6^A on TRAM2 which activates the Hippo pathway	[107]
Lung adenocarcinoma (LUAD)	Hippo pathway	↑METTL3	Unknown	m^6^A‐mediated upregulation of lncRNASNHG17 represses LATS2 modulating Hippo pathway	[106]
				m^6^A‐mediated circKRT17 stabilization increases YAP1 nuclear localization	[105]
Lung adenocarcinoma (LUAD)	Notch signaling	↑METTL3	YTHDF2	METTL3 modified TUSC7 transcript is degraded by YTHDF2 which eliminates TUSC7 inhibition of Notch signaling	[115]
Epithelial ovarian cancer (EOC)	NF‐κB signaling	METTL3	YTHDF1	m^6^A‐mediated RIPK4 mRNA stabilization and activation of NF‐κB	[116]
Ovarian cancer	cAMP‐dependent signaling	↑METTL3	Unknown	m^6^A‐mediated PTGER2 is upregulated conferring carboplatin resistance to cancer cells	[111]

The PI3K/Akt/mTOR pathway activation is frequently observed in various cancers and it is also associated with chemotherapeutic resistance. Deregulation of m^6^A modification plays a pivotal role in the activation of this signaling and ensuing cancer chemoresistance. In non‐small cell lung cancer, METTL3 upregulation causes elevated m^6^A modification and upregulation of Akt1, rendering the cancer cells resistant to cisplatin.^[^ ^108^ ^]^ PI3K/Akt activation and cisplatin resistance also occur in ovarian^[^ ^109^ ^]^ and bladder^[^ ^110^ ^]^ cancer. In ovarian cancer, m^6^A modification and stabilization of circPLPP4 sponges miR‐136 which upregulate phosphoinositide‐3‐kinase regulatory subunit 1 (PIK3R1) and activates PI3K/Akt and enhances cisplatin resistance.^[^ ^109^ ^]^ However, in bladder cancer, cisplatin insensitivity is due to low m^6^A reader YTHDC1 expression. YTHDC1 downregulation destabilizes PTEN, a PI3K/Akt suppressor. Activation of this pathway enhances DNA damage repair, apoptosis evasion, and cell cycle recovery thereby reducing cisplatin response in cancer cells.^[^ ^110^ ^]^ METTL3 upregulation is associated with carboplatin resistance in ovarian cancer via activation of cAMP‐dependent signaling. m^6^A modification of prostaglandin E receptor 2 (PTGER2) is upregulated in ovarian cancer; this is associated with self‐renewal and DNA damage repair capabilities in cancer cells leading to resistance to therapy.^[^ ^111^ ^]^

In epithelial ovarian cancer (EOC), ALKBH5 is upregulated; this is associated with cisplatin resistance via erasure of the m^6^A modification on JAK2 mRNA which protects it from YTHDF2‐mediated degradation. Elevated JAK2 consequently activates STAT3 protein and results in cisplatin resistance. ALKBH5 thus contributes to EOC cisplatin resistance via activation of the JAK2/STAT3 signaling pathway.^[^ ^112^ ^]^ FTO‐mediated m^6^A reduction and doxorubicin resistance via STAT3 activation also occur in breast cancer.^[^ ^58^ ^]^ In contrast, in pancreatic ductal adenocarcinoma (PDAC), ALKBH5 is downregulated and is associated with gemcitabine resistance. ALKBH5 erases m^6^A from 3′‐UTR of the Wnt inhibitory factor1 (WIF1) mRNA and promotes its transcription; this is then downregulated due to lack of ALKBH5 which in turn activates the Wnt signaling pathway.^[^ ^113^ ^]^

Activation of the MAPK/ERK pathway is another mechanism of malignancy in various types of cancer. In hepatocellular carcinoma, increased METTL3 expression enhances chemoresistance by m^6^A modification and overexpression of lncRNA DUXAP8. DUXAP8 sponges miR‐584‐5p to upregulate MAPK1 resulting in resistance to sorafenib.^[^ ^114^ ^]^ METTL3 contributed to erlotinib resistance in lung adenocarcinoma through eliminating suppression of Notch signaling by TUSC7. YTHDF2 recognizes the m^6^A modification on TUSC7, accelerating its degradation and eliminating TUSC7 suppression of the Notch signaling proteins; this results in erlotinib resistance.^[^ ^115^ ^]^ METTL3 also contributes to cisplatin resistance in EOC via activation of NF‐κB signaling. In EOC cells, oncogene receptor‐interacting protein kinase 4 (RIPK4) is upregulated due to METTL3‐mediated m^6^A modification and YTHDF1‐mediated mRNA stabilization. RIPK4 in turn activates NF‐κB which results in cisplatin resistance and tumor growth.^[^ ^116^ ^]^

## 6 m^6^A RNA Modification and Its Regulators in Radiation Therapy Resistance

Radiation therapy involves the use of ionizing radiation that causes DNA strand breaks culminating in cancer cell death. Radiation therapy targets cancer cells, minimizing the risk of normal cell death. It is frequently used prior to chemotherapy or immunotherapy to reduce the cancer cell mass.^[^ ^117^ ^]^ However, the development of resistance to radiation therapy, particularly due to enhanced DNA damage repair limits its effectiveness in cancer treatment, underscoring the need for a deeper understanding of the molecular mechanisms underlying such resistance. A number of recent studies demonstrate that m^6^A modification is a key player in radioresistance in cancer cells (**Table** **10**).^[^ ^118^ ^]^ m^6^A modification due to METTL3 upregulation in non‐small cell lung cancer is implicated in resistance to carbon‐ion radiotherapy. The DNA damage repair regulator and checkpoint protein, H2A histone family member X (H2AX), is the target of METTL3 which upon m^6^A methylation is upregulated; this leads to decreased sensitivity of cancer cells to carbon‐ion radiotherapy.^[^ ^119^ ^]^ METTL3 upregulation and increased m^6^A in lncRNA LINC00839 are associated with radiation resistance in GSCs. m^6^A‐modified LINC00839 is stabilized by YTHDF2 and upregulates LINC00839 which further activates the Wnt/β‐catenin signaling pathway and contributes to radioresistance.^[^ ^120^ ^]^ METTL3‐mediated m^6^A RNA methylation and stabilization of circular RNA circCUX1 contributes to radiotherapy resistance via its regulation of Caspase 1 expression in hypopharyngeal squamous cell carcinoma (HPSCC).^[^ ^121^ ^]^ Cervical cancer (CC) cells also exhibit resistance to radiation therapy in an m^6^A‐dependent manner. Hepatocyte nuclear factor 1‐alpha (HNF1α)‐induced YTHDF3 upregulation enhances RAD51 homolog 4 (RAD51D) translation due to m^6^A modification on the RAD51D transcript which results in its upregulation. RAD51D is a vital DNA repair protein that plays an important role in repairing the DNA strand break induced by ionizing radiation; its overexpression renders cervical cancer cells resistant to irradiation therapy.^[^ ^122^ ^]^ Another member of the methyltransferase complex, WTAP, induces breast cancer cell radioresistance by increasing Bcl‐2 expression. YTHDF1 enhances the translation of m^6^A‐modified Bcl‐2; this leads to the upregulation of this antiapoptotic protein in breast cancer cells and confers them with radioresistance.^[^ ^123^ ^]^ The m^6^A eraser, FTO, promotes radioresistance in nasopharyngeal carcinoma (NPC) cells through repression of radiation‐induced ferroptosis. Removal of m^6^A from OTUB1 (a critical DNA damage response regulator) transcript stabilizes it and induces resistance to irradiation.^[^ ^124^ ^]^ Another m^6^A eraser, ALKBH5, is upregulated in glioblastoma and associated with radiotherapy resistance via upregulation of DNA damage repair genes.^[^ ^125^ ^]^ In contrast, increased m^6^A levels contribute to radiotherapy resistance in NPC through activation of EGFR signaling. The m^6^A reader, YTHDC2, binds to the insulin‐like growth factor 1 receptor (IGF1R), promotes its translation, and activates IGF1R/Akt/S6 signaling; this leads to radiation resistance.^[^ ^126^ ^]^ m^6^A modification confers radiotherapy resistance to bone metastatic prostate cancer (mPCa) cells through the stabilization of enhancer RNA MLXIPe which, through its enhancer activity, promotes oncogenic PSMD9 transcription.^[^ ^127^ ^]^ m^6^A modification also confers radiation resistance to cancer cells indirectly via suppression of immune cell infiltration in the tumor microenvironment. m^6^A modification of bone morphogenetic protein and activin membrane‐bound inhibitor (BAMBI) in the MDSC causes its degradation via YTHDF2, allowing the MDSC to exert its suppressive effect on immune cell infiltration in the TME which leads to reduced sensitivity to ionizing radiation (IR).^[^ ^128^ ^]^

**Table 10:** m^6^A‐mediated radioresistance.

Cancer type	Molecular target	Writer/Eraser	m^6^A Reader	Mechanism	Reference
Glioma	LINC00839	↑METTL3	↑YTHDF2	m^6^A modification stabilizes and upregulates LINC00839 which activates Wnt/β‐catenin signaling leading to radiotherapy resistance	[120]
Non‐small cell lung cancer (NSCLC)	H2AX	↑METTL3	Unknown	METTl3‐mediated m^6^A modification upregulates H2AX which enhances DNA damage repair leading to carbon‐ion therapy resistance	[119]
Nasopharyngeal carcinoma (NPC)	OTUB1	↑FTO	Not involved	FTO demethylates OTUB1 causing its overexpression which inhibits radiation‐induced ferroptosis	[124]
Nasopharyngeal carcinoma (NPC)	IGF1R	Unknown	↑YTHDC2	m^6^A modified IGF1R is upregulated with activation of the IGF1R/Akt/S6 pathway leading to resistance	[126]
Bone metastatic prostate cancer (mPCa)	MLXIPe	Unknown	KHSRP	m^6^A modification of enhancer RNA MLXIPe is stabilized by KHSRP and upregulates PSMD9 to cause radioresistance	[127]
Cervical cancer (CC)	RAD51D	Unknown	↑YTHDF3	YTHDF3 increases RAD51D translation leading to DNA damage repair by irradiation	[122]
Breast cancer	Bcl‐2	↑WTAP	↑YTHDF1	Increased Bcl‐2 expression due to m^6^A modification results in decreased irradiation‐induced apoptosis	[123]
Hypopharyngeal squamous cell carcinoma (HPSCC)	circCUX1	↑METTL3	Unknown	m^6^A modification stabilizes circCUX1 which inhibits Caspase 1 expression and development of radioresistance	[121]
Glioblastoma	CHK1	↑ALKBH5	Not involved	ALKBH5 increases the expression of DNA damage repair genes leading to radioresistance	[125]

## 7 m^6^A Targeted Therapy: A Novel Approach for Overcoming Cancer Therapy Resistance

**Figure 4:**
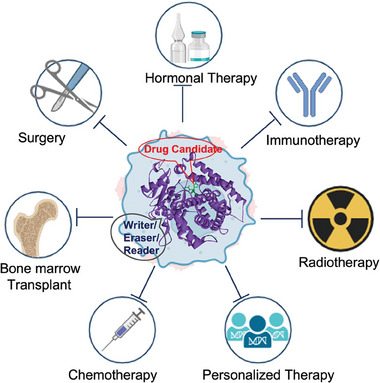
m^6^A RNA modification is associated with resistance development against conventional cancer therapeutic options. Specific inhibitors developed against different m^6^A regulators (writers, erasers or readers) could be a promising approach to overcoming resistance to cancer therapeutics.

The involvement of m^6^A modification and its regulators in therapeutic resistance to cancer is evidenced in numerous studies (**Figure** **4**). However, m^6^A‐based therapeutic intervention for cancer therapeutics is still in its infancy. A number of molecules have been tested for their therapeutic potential which were mostly targeted to various m^6^A regulators (writers, erasers and readers) (**Table** **11**).

**Table 11:** m^6^A‐targeted therapy.

Drug/Compound	Target m^6^A Regulator	Cancer/Cell Type	In vitro or in vivo effects	Reference
**Writers**				
Compound 2 & 7 (nucleotide analogs)	METTL3	N/A	Decreases m^6^A by mimicking substrate binding	[129]
UZH1a UZH1b	METTL3	Acute myeloid leukemia (AML) Osteosarcoma	Decreases m^6^A in tested cell lines	[130]
UZH2 (1,4,9‐triazapriro[5,5]undecan‐2‐one)	METTL3	Acute myeloid leukemia (AML) Prostate cancer	Decreases m^6^A in tested cell lines	[131]
STM2457	METTL3	Acute myeloid leukemia (AML)	Inhibition of METTL3 and reduction in m^6^A results in decreased tumor growth and cancer stem cell population	[132]
CDIBA (4‐[2‐[5‐chloro‐1‐(diphenylmethyl)−2‐methyl‐1H‐indol‐3‐yl]‐ethoxy]benzoic acid)	METTL3/METTL14 complex	Acute myeloid leukemia (AML)	m^6^A downregulation results in decreased proliferation of AML cells	[134]
Eltrombopag	METTL3/METTL14 complex	Acute myeloid leukemia (AML)	Antiproliferative effect against AML	[135]
Elvitegravir	METTL3	Esophageal squamous cell carcinoma (ESCC)	Decreased metastasis of ESCC	[136]
**Erasers**				
Rhein	FTO	Neuroblastoma	Inhibits FTO activity and increases m^6^A level	[137]
Ethyl ester form of meclofenamic acid MA2	FTO	Human HeLa cells	Increases m^6^A level in mRNA of HeLa cells	[138]
FB23 FB23‐2	FTO	Acute myeloid leukemia (AML)	Increases m^6^A and decreases proliferation of AML cells	[139]
Dac51	FTO	Melanoma Non‐small cell lung cancer (NSCLC)	Synergistic effect with anti‐PD‐L1 therapy resulting in decreased tumor growth	[140]
FTO‐02 FTO‐04	FTO	Glioblastoma	Increases m^6^A inhibits neurosphere formation and self‐renewal of GSCs	[141]
R‐2HG	FTO	Acute myeloid leukemia (AML) Glioma	FTO inhibition decreases MYC/CEBPA stability resulting in reduced cancer cell proliferation/survival	[33]
CS1 CS2	FTO	Acute myeloid leukemia (AML)	Decreases leukemia stem cells and overcomes immune check point blockade	[34]
Entacapone	FTO	N/A	Results in decreased body weight and fasting blood glucose levels in high‐fat diet‐induced obese mice (db/db mice) through inhibition of FTO	[142]
Nafamostat mesylate	FTO	N/A	Shows FTO inhibition in biochemical studies and in silico	[143]
Clausine E	FTO	N/A	Shows FTO inhibition in biochemical studies and in silico	[144]
2‐[(1‐hydroxy‐2‐oxo‐2‐phenylethyl)sulfanyl] acetic acid 4‐[(furan‐2‐yl)methyl]amino‐1,2‐diazinane‐3,6‐dione	ALKBH5	Leukemia cells	ALKBH5 inhibition and anti‐leukemic effect	[145]
20m	ALKBH5	HepG2 cells	Increases m^6^A levels in cells	[146]
DDO‐2728	ALKBH5	Acute myeloid leukemia (AML)	Inhibits ALKBH5 suppressed tumor growth	[147]
Ena15 Ena21	ALKBH5 FTO (Ena21)	Glioblastoma	Halts glioblastoma cell proliferation	[148]
**Readers**				
BTYNB (2‐[(5‐bromo‐2‐thienyl) methylene]amino benzamide)	IGF2BP1	Ovarian cancer Melanoma	Inhibits proliferation of cancer cells	[149]
		Leukemia cells	Decreases leukemia cell proliferation and induced differentiation	[150]
CWI1‐2	IGF2BP2	Acute myeloid leukemia (AML)	Anti‐leukemic effect in vitro and in vivo	[44]
JX5	IGF2BP2	T cell acute lymphoblastic leukemia (T‐ALL)	Reduces T‐ALL cell proliferation	[151]
AE‐848	IGF2BP3	Ovarian cancer	Shows antiproliferative effect against ovarian cancer and helps overcome immune suppression	[152]

### 7.1 Targeting m^6^A Writers

METTL3 is the main catalytic component of the m^6^A methyltransferase complex and is associated with chemoresistance in many cancer types. Therefore, METTL3 could be a primary target for overcoming resistance to therapeutic agents. Initially, two small molecules were discovered as METTL3 inhibitors using molecular docking based on the X‐ray crystallographic structure of the METTL3 binding site; both of these small molecules were nucleotide analogs.^[^ ^129^ ^]^ A second structure‐based inhibitor design by the same group gave rise to UZH1a and its enantiomer UZH1b. UZH1a is 100 times more potent than UZH1b in terms of METTL3 inhibition and m^6^A reduction.^[^ ^130^ ^]^ With a goal of discovering more potent METTL3 inhibitors, this group developed UZH2 (1,4,9‐triazapriro[5,5]undecan‐2‐one) which has higher potency and more favorable ADME (absorption, distribution, metabolism, and excretion) features.^[^ ^131^ ^]^ Another group developed a potent METTL3 inhibitor, STM2457, and tested its efficacy against leukemia. STM2457 shows promising effects in AML cells, reducing tumor growth by inducing cancer cell apoptosis. This novel compound also decreases cancer stem cell populations (nanomolar concentration) and also has a favorable ADME profile.^[^ ^132^ ^]^ The potential of targeting the METTL3 in cancer therapy encouraged pharmaceutical industry research in specific inhibitors, resulting in development of a few small molecular inhibitors, e.g., by Accent Therapeutics (patents WO 2021079196 A2, WO 2021081211 A1, and WO 2022081739 A1) and Storm Therapeutics (patents WO 2020201773 A1, WO 2022074379 A1, and WO 2022074391 A1); these show promise as novel anticancer therapeutics.^[^ ^133^ ^]^ An allosteric inhibitor of METTL3‐METTL14 methyltransferase complex, 4‐[2‐[5‐chloro‐1‐(diphenylmethyl)−2‐methyl‐1H‐indol‐3‐yl]‐ethoxy]benzoic acid (CDIBA) shows promise with its antiproliferative effect in AML cells (micromolar concentrations).^[^ ^134^ ^]^ A thrombopoietin receptor antagonist (TPO‐R), Eltrombopag which was approved by USFDA to treat anemia, is an allosteric antagonist of METTL3‐METTL14 complex that also exhibits an antiproliferative effect in AML.^[^ ^135^ ^]^ The antiviral drug, elvitegravir, accelerates proteasomal degradation of METTL3 and suppresses metastatic spread of esophageal squamous cell carcinoma (ESCC).^[^ ^136^ ^]^ METTL3 is therefore a promising target for novel therapeutics to treat cancer or to overcome resistance.

### 7.2 Targeting m^6^A Erasers

Similar to m^6^A methyltransferases, upregulation of m^6^A demethylases occurs in various cancer types and is therefore also of interest to drug discovery scientists. The natural product, rhein, competitively binds to the active site of FTO resulting in a considerable FTO inhibitory effect.^[^ ^137^ ^]^ An ethyl ester form of a non‐steroidal anti‐inflammatory drug, meclofenamic acid (MA), MA2 is an FTO inhibitor with better selectivity compared to another demethylase, ALKBH5.^[^ ^138^ ^]^ Based on the results of the structure‐based inhibitor design of MA, the same group developed two more selective FTO inhibitors FB23 and FB23‐2. These were tested on AML cells, and showed promising results in suppressing proliferation and proapoptotic effects.^[^ ^139^ ^]^ Further optimization of FB23 and FB23‐2 led to the development of a more potent FTO inhibitor, Dac51, which enhances T cell infiltration in the tumor microenvironment and therefore suppressing melanoma and NSCLC growth. Dac51 also has a synergistic effect with anti‐PD‐L1 immunotherapy in halting tumor growth, showing its promise to overcome cancer cell resistance to anti‐PD‐L1 therapy.^[^ ^140^ ^]^ Meanwhile, Huff et al. developed two additional selective FTO inhibitors, FTO‐02 and FTO‐04, using a structure‐based drug design approach. FTO‐04 has an inhibitory effect on glioblastoma, blocking neurosphere formation and self‐renewal capabilities of GSCs and therefore demonstrating its potential to overcome chemoresistance.^[^ ^141^ ^]^ The enantiomer of 2‐hydroxyglutarate (R‐2HG), an oncometabolite, also has FTO inhibitory properties. R‐2HG is the product of a mutant IDH gene that exhibits tumor suppression and has an antitumor effect in leukemia and glioma via blockade of FTO‐mediated demethylation of and degradation of MYC/CEBPA transcripts.^[^ ^33^ ^]^ Based on structure‐based virtual screening and testing in AML cell models, two other FTO inhibitors CS1 and CS2 were discovered. Both of these compounds show high efficacy for FTO inhibition as well as potent antitumor effects via suppression of self‐renewal capability in leukemia stem/initiating cells thereby overcoming immune evasion. CS1/2 thus shows promise to overcome AML resistance due to stem cell generation or immune checkpoint blockade.^[^ ^34^ ^]^ Through structure‐based virtual screening of FDA‐approved anti‐Parkinson's drug, entacapone, researchers observed inhibition of FTO (micromolar concentration), the anticancer effect of which is yet to be tested.^[^ ^142^ ^]^ Nafamostat mesylate, a potent protease inhibitor, used for pancreatitis and disseminated intravascular coagulation has an anticancer effect as an FTO inhibitor.^[^ ^143^ ^]^ A carbazole alkaloid, Clausine E, has antiproliferative effects in colorectal cancer cells; it is also an FTO inhibitor.^[^ ^144^ ^]^ Another m^6^A demethylase, ALKBH5, is implicated in cancer development and chemoresistance in multiple studies. ALKBH5 inhibitors have also been developed and tested against cancer cells. A high‐throughput virtual screening identified two compounds, 2‐[(1‐hydroxy‐2‐oxo‐2‐phenylethyl)sulfanyl]acetic acid and 4‐[(furan‐2‐yl)methyl]amino‐1,2‐diazinane‐3,6‐dione, as ALKBH5 inhibitors which have an antiproliferative effect against three leukemia cell lines (micromolar concentrations).^[^ ^145^ ^]^ Through structure‐activity relationship analysis, another potent ALKBH5 inhibitor (20m) has been developed which contains a 1‐aryl‐1H‐pyrazole scaffold.^[^ ^146^ ^]^ Structure‐based virtual screening gave rise to a “pyrazolo[1,5‐a]pyrimidine” derivative ALKBH5 inhibitor, DDO‐2728. In tests in human AML cells, investigators observed highly potent and selective ALKBH5 inhibition; through inhibition of cell cycle progression, it suppresses tumor growth.^[^ ^147^ ^]^ Two additional ALKBH5 inhibitors were discovered through a high‐throughput screening from a small molecule library by another group‐ Ena15 and Ena21. Ena15 shows selective inhibition of ALKBH5 while Ena21 shows little FTO inhibitory effect. Both compounds halt the proliferation of glioblastoma cells in vitro experiments, highlighting their potential as a novel therapeutic agent for cancer.^[^ ^148^ ^]^

### 7.3 Targeting m^6^A Readers

m^6^A readers have been targeted for anticancer therapy due to their protumorigenic role and involvement in inducing cancer therapy resistance. Small molecular library screening in search of an IMP1 (IGF2BP1) inhibitor revealed a small molecule 2‐[(5‐bromo‐2‐thienyl) methylene]amino benzamide (BTYNB) with potent IMP1 inhibitory effect which inhibited proliferation of ovarian cancer and melanoma cells.^[^ ^149^ ^]^ BTYNB reduces the proliferation of leukemia cells and induces their differentiation.^[^ ^150^ ^]^ Increased IGF2BP2 in AML cells is associated with tumorigenesis and self‐renewal of leukemia stem cells. Inhibition of IGF2BP2 with a novel small molecule, CWI1‐2, has an antitumor effect in AML cells.^[^ ^44^ ^]^ JX5, another small molecule inhibitor of IGF2BP2 acts against T‐ALL cell proliferation.^[^ ^151^ ^]^ An inhibitor of another m^6^A reader, IGF2BP3, shows promise in ovarian cancer treatment. This small molecular inhibitor, AE‐848, along with its antiproliferative effect in ovarian cancer contributes to overcoming the immunosuppressive microenvironment by polarizing the tumor‐associated macrophages toward M1 type and inducing proinflammatory cytokines release (IFN‐γ and TNF‐α). AE‐848 through its inhibition of m^6^A reader, IGF2BP3, may contribute to halting tumor growth as well as to overcoming therapeutic resistance.^[^ ^152^ ^]^

## 8 Conclusion and Future Perspectives

The emergence of resistance to anticancer therapies poses significant challenges in cancer treatment resulting in therapeutic failure; it is responsible for cancer recurrence and higher mortality rates. Cancer cells have developed resistance to most commonly used therapeutics, necessitating multidrug treatment regimens. These combinations, enhance treatment‐associated toxicities and long‐term disabilities and also increase the financial burden for patients, even though complete remission is uncertain. Hence, understanding the molecular mechanism(s) underlying the development of resistance is critical to therapeutic success and reducing patient mortality. Epitranscriptomic RNA m^6^A modification has emerged as an important factor in the development of resistance to diverse classes of chemotherapeutic, immunotherapeutic, and targeted therapies as well as radiotherapeutic agents. m^6^A modification and its regulatory machinery are implicated in resistance to more than one class of chemotherapeutics and radiotherapy as reported in multiple studies.^[^ ^153^ ^]^ In one study Taketo et al. showed that METTL3 upregulation confers pancreatic cancer cells' resistance to gemcitabine, 5‐FU, cisplatin, and radiation therapy^[^ ^153a^ ^]^ while Liu et al. reported on the role of WTAP in the development of resistance in gastric cancer cells to cisplatin, cyclophosphamide, and irradiation.^[^ ^153b^ ^]^ m^6^A RNA modification and its regulators involved in resistance to therapy hold promise as novel targets in overcoming multidrug resistance. If effective, this has the advantage of reducing the adverse effects of multidrug regimens and financial burden. If used in combination with conventional anticancer drugs, these have the potential to improve efficacy. There are, however, multiple challenges to targeting the m^6^A regulatory process in oncotherapy. Inhibitor development for m^6^A regulators is still in its infancy. Numerous molecules have been developed as regulators of m^6^A writers, erasers, and readers in silico, based on their crystallographic or other structural features; however, it is uncertain how many will actually show optimal inhibition in vitro or in vivo. Although some newly synthesized inhibitors showed promising results in vitro and to some extent in vivo in animal experiments, they are yet to be tested in preclinical or clinical trials. Another issue is ensuring target specificity; this is critical because lack of specificity is a primary cause of adverse or toxic effects in humans. m^6^A regulators incorporate and recognize or remove m^6^A modification marks from their target transcripts causing up‐ or downregulation in cancer cells. There may be more than one target transcript being modified by a particular m^6^A regulator; these target transcripts may play vital biological roles in otherwise normal cells. Inhibition of the regulator may produce an undesired effect in the patients. Therefore, specific inhibition of the regulator in cancerous cells and not in normal cells is of utmost importance. A cancer‐associated molecular target may have more than one m^6^A modification site on its transcript. Determination of the causal relationship between each m^6^A modification site and its proto‐ or antioncogenic function in cancer cells will facilitate the development of more targeted specific therapies. In recent years, scientists developed techniques to engineer RNA molecules for installing and erasing the m^6^A marks on target transcripts. Most of these techniques are based on modified CRISPR‐Cas genome editing methods for RNA engineering, incorporating a catalytic domain from an RNA methyltransferase or a demethylase.^[^ ^154^ ^]^ Using these techniques, Ying et al. observed a protumorigenic role of m^6^A‐modification at 3′‐UTR of CUB domain‐containing protein 1 (CDCP1) in bladder cancer.^[^ ^155^ ^]^ They also developed a multisite RNA‐m^6^A editor and utilized this to manipulate and study the tumorigenic role of m^6^A modification at four sites of integrin α6 (IGTA6). They site‐specifically erased m^6^A from IGTA6 transcript and restricted the proliferation and migration of bladder cancer cells.^[^ ^156^ ^]^ These studies show promise for the use of RNA‐m^6^A engineering as a diagnostic and therapeutic tool for target‐specific anticancer therapy development.

There are seemingly contradictory reports regarding the involvement of m^6^A regulators in carcinogenesis or the development of resistance. Some researchers report overexpression of writers such as METTL3 or METTL14 to be an underlying reason for the development of resistance in a particular cancer type while others report the opposite, i.e., upregulation of m^6^A erasers FTO or ALKBH5 and resultant low m^6^A levels as the underlying cause. One explanation for this could be the differential oncogenic targets by different regulators in different individuals. Investigators need to ascertain which m^6^A regulator is involved before any agent is selected for targeting. Recent studies show that targeting m^6^A modification regulators enhances the sensitivity of some of the clinically used anticancer drugs showing the promise of exploiting this RNA modification mechanism to reverse the resistance and sensitize refractory cancer cells to therapy.

## Conflict of Interest

The authors declare no conflict of interest.

## Author Contributions

M.B.U., Z.W., and C.Y. designed the study. M.B.U. drafted the manuscript. M.B.U., Z.W., and C.Y. reviewed and revised the manuscript. All authors read and approved the final manuscript.
